# Expression and Secretion of Circular RNAs in the Parasitic Nematode, *Ascaris suum*

**DOI:** 10.3389/fgene.2022.884052

**Published:** 2022-05-31

**Authors:** Sarah J. Minkler, Hannah J. Loghry-Jansen, Noelle A. Sondjaja, Michael J. Kimber

**Affiliations:** Department of Biomedical Sciences, College of Veterinary Medicine, Iowa State University, Ames, IA, United States

**Keywords:** *Ascaris suum*, circular RNA, extracellular vesicles, ivermectin, parasite

## Abstract

Circular RNAs (circRNAs) are a recently identified RNA species with emerging functional roles as microRNA (miRNA) and protein sponges, regulators of gene transcription and translation, and modulators of fundamental biological processes including immunoregulation. Relevant to this study, circRNAs have recently been described in the parasitic nematode, *Haemonchus contortus*, suggesting they may have functionally important roles in parasites. Given their involvement in regulating biological processes, a better understanding of their role in parasites could be leveraged for future control efforts. Here, we report the use of next-generation sequencing to identify 1,997 distinct circRNAs expressed in adult female stages of the gastrointestinal parasitic nematode, *Ascaris suum.* We describe spatial expression in the ovary-enriched and body wall muscle, and also report circRNA presence in extracellular vesicles (EVs) secreted by the parasite into the external environment. Further, we used an *in-silico* approach to predict that a subset of *Ascaris* circRNAs bind both endogenous parasite miRNAs as well as human host miRNAs, suggesting they could be functional as both endogenous and exogenous miRNA sponges to alter gene expression. There was not a strong correlation between *Ascaris* circRNA length and endogenous miRNA interactions, indicating *Ascaris* circRNAs are enriched for *Ascaris* miRNA binding sites, but that human miRNAs were predicted form a more thermodynamically stable bond with *Ascaris* circRNAs. These results suggest that secreted circRNAs could be interacting with host miRNAs at the host-parasite interface and influencing host gene transcription. Lastly, although we have previously found that therapeutically relevant concentrations of the anthelmintic drug ivermectin inhibited EV release from parasitic nematodes, we did not observe a direct effect of ivermectin treatment on *Ascaris* circRNAs expression or secretion.

## Introduction

Circular RNAs (circRNAs) are a species of long, noncoding RNA that do not contain an open 5′ or 3’ end but instead form a circular structure that is more stable than linear RNA species (Enuka et al., 2016). The majority of circRNAs are approximately 1,500 nucleotides (nt) or less and have a median length of 550 nt (Zheng et al., 2016; Ding et al., 2018). circRNAs were first discovered through electron microscopy imaging of HeLa cells, CV-1 cells (monkey kidney cell line), and Chinese hamster ovary cells (Hsu and Coca-Prados, 1979) and initially thought to be the product of nontraditional splicing, forming “scrambled exons” with no real function or significance (Nigro et al., 1991). With advancements in the sensitivity of high throughput sequencing and data analysis pipelines, the complexity of the circRNA complement has been recognized, validated, and shown to be functionally active in a variety of species including humans (Memczak et al., 2013; Guo et al., 2014), mice (Memczak et al., 2013), insects (Westholm et al., 2014), plants (Zhang et al., 2020), fungi (Shao et al., 2019), and, germane to the current study, the model nematode *Caenorhabditis elegans* (Memczak et al., 2013; Ivanov et al., 2015; Cortés-López et al., 2018). The recognition that circRNAs are expressed in *C. elegans* has recently seeded their discovery in parasitic nematodes, specifically, the small ruminant gastrointestinal parasitic nematode, *Haemonchus contortus* (Zhou et al., 2021).

**FIGURE 1:**
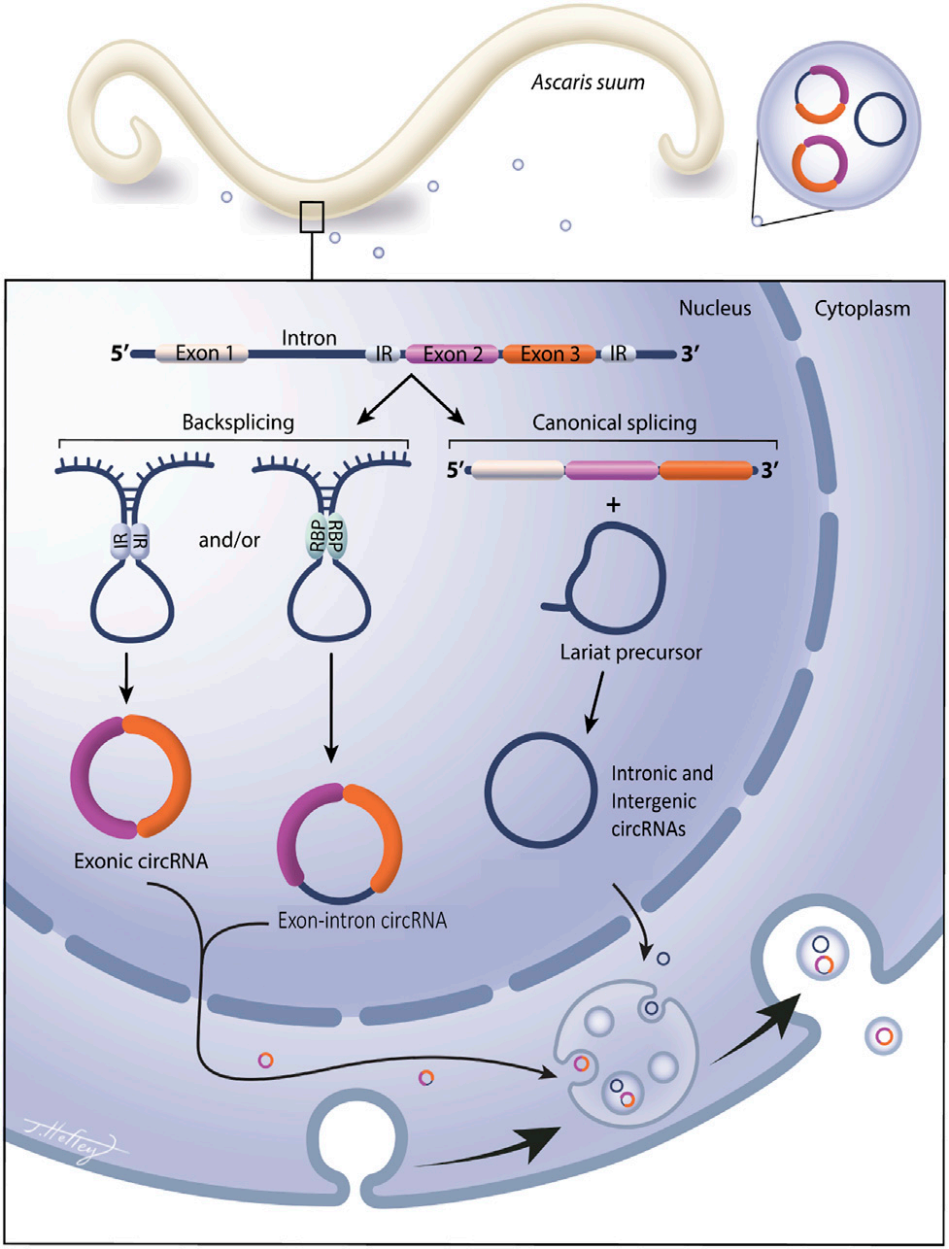
Circular RNAs are expressed in the gastrointestinal parasitic nematode *Ascaris suum* and secreted into the host environment via extracellular vesicles. Circular RNA (circRNA) are covalently closed circular RNA rings, with no open 5′ or 3′ ends. They do not contain a polyA tail or a 5′ cap and are extremely stable and less prone to degradation than linear RNA species. Exonic circRNAs contain only exonic RNA, intergenic circRNAs and intronic circRNAs contain only introns. Exonic, exon-intron circRNAs are generated from a back splicing event where inverted repeats (IR) or RNA binding proteins (RBP) form a semi-closed covalent ring, allowing for downstream splice donor site to attack upstream slice acceptor site, forming the closed circRNA structure. Intronic and intergenic circRNAs are formed from lariat precursor molecules during linear splicing. Our data show exonic, intergenic and intronic circRNAs are expressed in *Ascaris* tissue and are also packaged into parasitic EV cargo for secretion into the external environment.

The biogenesis of circRNAs is summarized in Figure 1. Exonic (containing only exons) and exon-intron circRNAs (containing both exons and introns) are formed when pre-mRNA transcripts undergo a back-splicing event where a downstream splice donor site attacks an upstream splice acceptor site (Memczak et al., 2013). These splice sites are brought together through intron looping that is facilitated by inverted repeat base pairing (Ivanov et al., 2015) or via pairing of RNA-binding proteins (RBPs) (Conn et al., 2015; Errichelli et al., 2017). Intergenic circRNAs are formed in a similar manner as exonic circRNAs, but unlike exonic circRNAs, intergenic circRNAs contain two intron circRNA fragments that are surrounded by GT-AG sites (Geng et al., 2018). Distinct from this process, intronic circRNAs (containing intronic RNA only) are formed from lariat precursors during linear splicing that evaded debranching and remained in a circular structure, avoiding degradation (Kristensen et al., 2019). A fundamental function of circRNAs is the regulation of gene expression, which is accomplished through multiple pathways. The most recognized is that circRNAs act as miRNA sponges, binding multiple miRNAs and influencing gene expression by reducing miRNA bioavailability. This property of miRNA binding was first discovered in mice by Hansen et al. (2013) who found that CDR1as could bind murine miRNAs and modify miRNA biological functions as a result. Numerous miRNA binding sites have also been found in *Drosophila* circRNAs (Westholm et al., 2014) in support of this role. In addition, circRNAs can also promote gene transcription through interactions with RNA polymerase II and U1 snRNP in the promoter region of a gene (Li Z. et al., 2015), and in some instances, circRNAs can also be translated into proteins but the function of circRNA translated proteins remains largely unexplored (Pamudurti et al., 2017).

Currently, there is sparse data on the expression of circRNAs in nematodes. circRNAs have been identified in *C. elegans* (Memczak et al., 2013; Ivanov et al., 2015; Cortés-López et al., 2018) with the first descriptive study of a nematode circRNA complement based on *H. contortus* recently emerging (Zhou et al., 2021). These manuscripts focus on the presence of circRNAs in these two clade V nematode species but do not give much insight into the functional significance of circRNAs in worms. Here we describe the spatial expression of circRNAs in the clade III nematode *Ascaris suum. A. suum* is a large gastrointestinal parasite that primarily infects swine but has also been shown to infect humans. There are studies suggesting that *A. suum* and *Ascaris lumbricoides*, a human gastrointestinal nematode, are the same species due to cross infections between humans and pigs (Leles et al., 2012) and similarities in nucleic acid profiles (Nejsum et al., 2005; Shao et al., 2014). Infections with *A. suum* in pigs lead to decreased farming productivity, and carries negative economic impacts including reduced animal growth, losses of meat product from contamination, treatment costs, and co-infections with other pathogens (Thamsborg et al., 2013). In humans, 807 million -1.2 billion people are infected with *Ascaris* worldwide (Centers for Disease Control, 2020). Infections with *Ascaris* can lead to gastrointestinal obstructions, anemia, diarrhea, hepatobiliary, and pancreatic syndromes. There is a disproportionate number of infections in children, which can produce malnutrition and cognitive impairment (Bethony et al., 2006).

circRNAs are known to be secreted into the extracellular environment via extracellular vesicles (EVs) (Lasda et al., 2016), but have not been shown to be secreted in parasitic nematode EVs. Our laboratory and others, have previously shown that parasitic nematodes secrete EVs and that these EVs contain small RNA species (Buck et al., 2014; Hansen E. et al., 2019; Zamanian et al., 2015; Gu et al., 2017) but the presence of circRNAs has not been demonstrated in parasitic nematode EVs to date. We hypothesized that *A. suum* expresses endogenous circRNAs that may function as miRNA sponges. Further, that a cohort of these circRNAs would be secreted and that these secreted circRNAs could interact with host miRNAs to have an impact at the host-parasite interface. To investigate these hypotheses, we collected tissues from *A. suum* adult female parasites and used next-generation sequencing to describe the endogenous circRNA complement. We then tested for the presence of secreted circRNAs within *A. suum* EVs. We found a broadly distributed circRNA expression pattern in body wall muscle and ovarian tissue. Select circRNAs were also found to be secreted in EVs and this is the first study to describe this mechanism in parasitic helminths. Predicted binding of both endogenous and exogenous circRNAs to host and *Ascaris* miRNAs led to the hypothesis that circRNAs function as miRNAs sponges. These results suggest that circRNAs may function as miRNA sponges within *Ascaris* but, when secreted*,* may bind to host miRNAs and therefore influence host gene expression at the host-parasite interface.

## Materials and Methods

### Parasite Culture and Sample Collection

Healthy, live, adult female *Ascaris suum* were collected from swine hosts from an abattoir in Marshalltown, Iowa, United States. These parasites were thoroughly washed multiple times in *Ascaris* Ringer’s Solution (ARS) [(13.14 mM NaCl, 9.67 mM CaCl_2_, 7.83 mM MgCl_2_, 12.09 mM Tris, 99.96 mM sodium acetate, 19.64 mM KCl) with gentamycin (100 μg/ml), ciprofloxacin hydrochloride (20 μg/ml), penicillin (10,000 units/ml), streptomycin (10,000 μg/ml), and amphotericin B (25 μg/ml) at pH 7.87 (all Sigma Aldrich, St Louis, MO)] and then incubated at 35°C. The following day, parasites were checked visually for signs of bacterial or fungal contamination, and worms were discarded if present. Worms tissue and EVs were collected on the second day of culture to allow for an overnight acclimation period and to limit negative impact on gene expression. Adult female parasites were chosen due to their large size, facilitating the ease of collection material for RNA extraction. To obtain tissue for RNA isolation, worms were cut along the ventral midline and the ovaries were gently removed for excision. Tissue was collected proximal to the bifurcation of the ovaries and rinsed with fresh ARS. Given the location of ovarian tissue collection, we acknowledge residual embryonated egg material may be present and circRNA data should be interpreted with that in mind. Body wall tissue was collected directly anterior to the genital aperture and musculature was scraped from the underlying cuticle with a single-edge razor blade. Approximately 200 mg of body wall muscle and ovary tissue samples were obtained in each sample isolation. Tissues were either used for immediate RNA extraction or stored at -80°C until use.

### Drug Treatment

Individual worms were treated with 0.1 µM or 1 µM (final concentration) of ivermectin, diethylcarbamazine, or levamisole (all Sigma-Aldrich) for 24 h in 100 ml culture media in sterile 250 ml Erlenmeyer flasks. Drug concentrations were prepared from stock solutions dissolved in dimethyl sulfoxide (DMSO, Sigma-Aldrich). Conditioned media from drug treated and 0.1% DMSO vehicle control worms was collected after the 24-h time period and retained for downstream analysis. Body wall muscle and ovary-enriched tissue samples were collected from these parasites as described for immediate RNA extraction or storage at -80°C until use.

### EV Isolation and Quantification

EVs were collected as previously described using differential ultracentrifugation (Zamanian et al., 2015; Harischandra et al., 2018; Loghry et al., 2020). Media was filtered through 0.2 µm PVDF vacuum filters (Sigma-Aldrich) and centrifuged at 120,000 x g for 90 min at 4°C. The supernatant was decanted, and pellets were filtered through a PVDF 0.2 µm syringe filter (GE Healthcare, Chicago, IL) and centrifuged further at 186,000 x g for 2 hours at 4°C. EV samples were then resuspended to 500 µl in dPBS (Thermo Fisher Scientific, Waltman MA) and stored at -80°C until use.

EV quantification and size determination were performed using nanoparticle tracking analysis (NTA; Nano-Sight LM10, Malvern Instruments, Malvern, United Kingdom). EV imaging was performed using transmission electron microscopy. A 2 µl aliquot of isolated EV preparation was placed onto a carbon film grid for 1 min. The drop was wicked to a thin film and 2 µl of uranyl acetate (2% w/v final concentration) was immediately applied for 30 s, wicked, and allowed to dry. Images were taken using a 200kV JEOL 2100 scanning and transmission electron microscope (Japan Electron Optics Laboratories, LLC, Peabody, MA) with a Gatan OneView camera (Gatan, Inc. Pleasanton, CA).

### Circular RNA Isolation

Total RNA was extracted from adult female *A. suum* body wall and ovary tissues using a two-step process. First, worm tissues were homogenized in TRIzol reagent (Life Technologies, Carlsbad, CA) according to manufacturer recommendations. Following RNA isolation in TRIzol, total RNA was further purified using the miRNeasy Mini kit (QIAGEN, Hilden, Germany), following the manufacturer’s protocol and assessed for purity and quantified using a NanoVue spectrophotometer (General Electric, Boston, MA). Similarly, total RNA was extracted from EV enriched samples isolated from conditioned media using the miRNeasy Micro kit (QIAGEN), again following the manufacturer’s protocol. Total RNA from EV isolation supernatants (i.e. EV-depleted media) was extracted using Zymo ZR urine RNA isolation kit (Zymo Research, Irvine, CA), following manufacturer’s instructions. All linear RNA was subsequently removed from each of these total RNA preparations by RNase R digestion (RNase R was provided by the Singh Laboratory, Iowa State University). 10 Units of RNase R was used for each reaction along with 2 µg total RNA. Reactions were incubated at 37°C for 45 min followed by heat inactivation at 65°C for 20 min circRNA was then stored at -80°C until use. circRNA samples were sent to LC Sciences (Houston TX, USA) for sequencing or transcribed into cDNA for qPCR validation.

### CircRNA-Seq Library Preparation

CircRNA quality was assessed with a Bioanalyzer 2100 and RNA 6000 Nano LabChip Kit (Agilent, CA, USA), allowing a minimum RNA integrity number (RIN) of 7 (Schroeder et al., 2006) before fragmentation using NEBNext^®^ Magnesium RNA Fragmentation Module (NEB, Ipswich, MA) into short fragments using divalent cations under high temperature. The cleaved RNA fragments were then reverse-transcribed to create the cDNAs using SuperScript™ II Reverse Transcriptase (Thermo Fisher Scientific), which were next used to synthesize U-labeled second-stranded DNAs with *E. coli* DNA polymerase I (NEB), RNase H (NEB) and dUTP Solution (Thermo Fisher). An A-base was added to the blunt ends of each strand, preparing them for ligation to the indexed adapters. Each adapter contains a T-base overhang for ligating the adapter to the A-tailed fragmented DNA. Single- or dual-index adapters were ligated to the fragments, and size selection (300-600bp) performed with AMPureXP beads (Beckman Coulter, Brea, CA). After the heat-labile UDG enzyme (NEB) treatment of the U-labeled second-stranded DNAs, the ligated products were amplified with PCR by the following conditions: initial denaturation at 95°C for 3 min; eight cycles of denaturation at 98°C for 15 s, annealing at 60°C for 15 s, and extension at 72°C for 30 s; and then final extension at 72°C for 5 min. The average insert size for the final cDNA library was 300 ± 50 bp. Finally, 2 × 150bp paired-end sequencing (PE150) was performed on an Illumina Novaseq™ 6000 (Illumina) following the vendor’s recommended protocol.

### CircRNA Assembly

Cutadapt (Martin, 2011) and custom perl scripts were used to remove adaptors, low quality bases and undetermined bases, followed by quality assessments with FastQC (Andrews, 2010). Bowtie2 (Langmead & Salzberg, 2012) and Tophat2 (Kim et al., 2013) were used to map reads to the genome of *A. suum* (Wang J. et al., 2017) (Accession number: PRJNA62057; AGO1), with remaining unmapped reads remapped to the genome using Tophat-fusion (Kim & Salzberg, 2011). Dual *de novo* assemblies of circular RNAs were performed with CIRCExplorer (Zhang et al., 2014; Zhang et al., 2016), one with Bowtie2 and Tophat2 mapped reads and another with Tophat-fusion back-spliced reads. Since samples were not prepared simultaneously, the distinct circRNA assemblies were concatenated and filtered for uniqueness with duplicated sequences being removed.

### Analysis of circRNA-Seq Expression Data

Sequenced RNA reads (SRR) (SRR15295818-SRR15295823) were aligned to circRNAs from both body wall and ovary-enriched tissues, and to the *A. suum* genome (PRJNA62057), to reduce bias by avoiding creation of mapping reads that are artifacts from flawed methodology (Wang L. et al., 2017) using Hisat 2.2.0 (Kim et al., 2019). Samtools 1.10 (Li et al., 2009) was used to convert sam alignments to bam alignment files to map RNA sequencing reads to the *A. suum* genome and the three circRNA samples. Mapping statistics were assessed using Picard 2.17.0 (Institute, 2019). Read counts were taken using featureCounts from the Subread package 1.6.0 (Liao et al., 2014). Differential expression was assessed using DESEQ2 1.20.0 (Love et al., 2014), with both unique and multiple mapping reads considered in separate comparisons. Differentially expressed circRNAs were subjected to GO and KEGG enrichment analyses using clusterProfiler (Yu et al., 2012) and Ontologizer (Bauer et al., 2008). The number of exonic, intergenic, and intronic circRNAs were compiled from sequencing data and visualized using GraphPad Prism version 9.2.0 (GraphPad Software Inc., San Diego, CA). Notes and scripts used to produce expression analysis are available at https://github.com/ISUgenomics/Kimber. Raw data and circRNA sequences can be viewed using bio-project number PRJNA750737 with SRA numbers, SRR15295818 - SRR15295823.

### qRT-PCR circRNA Validation

Validation that circRNAs identified using circRNA-seq are expressed in *Ascaris* tissue or EV enriched samples was performed using quantitative real-time PCR (qRT-PCR). RNase R treated RNA (generated as described above) was reversed-transcribed to cDNA with random hexamers using Invitrogen SuperScript III First Strand Synthesis System (Thermo Fisher Scientific) according to the manufacturer’s instructions. A total of 10 sets of divergent primers targeting distinct circRNAs were designed to back-splice junction sites (Supplementary Table S2) and qRT-PCR was performed using Power Up Sybr Green Master Mix according to the manufacturer’s protocol (Thermo Fisher Scientific). Conditions for qRT-PCR were as follows: 2 min at 50°C, 2 min at 95°C, then 40 cycles of 15 s at 95°C, 15 s at 55–60°C, and 1 min at 72°C. CircRNA abundance was quantified by extrapolating qRT-PCR C_T_ values from exogenous (spiked in) *Homo sapiens* actin alpha cardiac muscle one RNA (GenBank accession number: NM_005159). This exogenous spike in RNA was generated using MEGAScript T7 transcription kit (Thermo Fisher Scientific) following manufacturer’s protocols corresponding to 517 bp–1,165 bp of the published transcript (primer sequences are detailed in Supplementary Table S2). The exogenous spike in RNA standard curve analysis was created using Graph Pad Prism version 9.2.0 (GraphPad Software Inc.) using a nonlinear second-degree polynomial, least squares fit. circRNA expression X values were interpolated to quantify the concentration of circRNA in each tissue type and EVs.

### CircRNA-miRNA Interactions

Two complementary programs were used to predict miRNAs and circRNA interactions: miRanda (https://github.com/hobywan/miranda) and Targetscan (Agarwal et al., 2018) using the identified *Ascaris* circRNA sequences and miRNA datasets for human and *A. suum* downloaded from miRbase.org (Release 22.1) (Kozomara and Griffiths-Jones, 2011). Within these programs, a higher miRanda free energy (200–140) and lower Targetscan score (-0.13–0) were used to assign interaction confidence. The number of *Ascaris* and human miRNA interactions for each *Ascaris* circRNA was totaled and visualized using GraphPad Prism (GraphPad Software Inc.). Similarly, *Ascaris* and human miRNAs with high numbers of predicted *Ascaris* circRNA binding partners were also collated and visualized using GraphPad Prism (GraphPad Software Inc.).

### Statistical Analysis

Drug treated tissues and EV circRNA expression levels were calculated from qRT-PCR C_T_ values using 2^−ΔΔCq^ (Livak & Schmittgen, 2001). Following fold change analysis, the data was log (2) transformed using GraphPad Prism (GraphPad Software Inc.). To compare circRNA concentrations between treatments and samples, a two-way ANOVA with multiple comparisons (GraphPad Software Inc.) was used with a *p*-value less than 0.05 being considered significant. Each N number represented a new and biologically distinct batch of worms, with an individual no-treatment control for each batch.

## Results

### A. suum circRNA Complement

**FIGURE 2:**
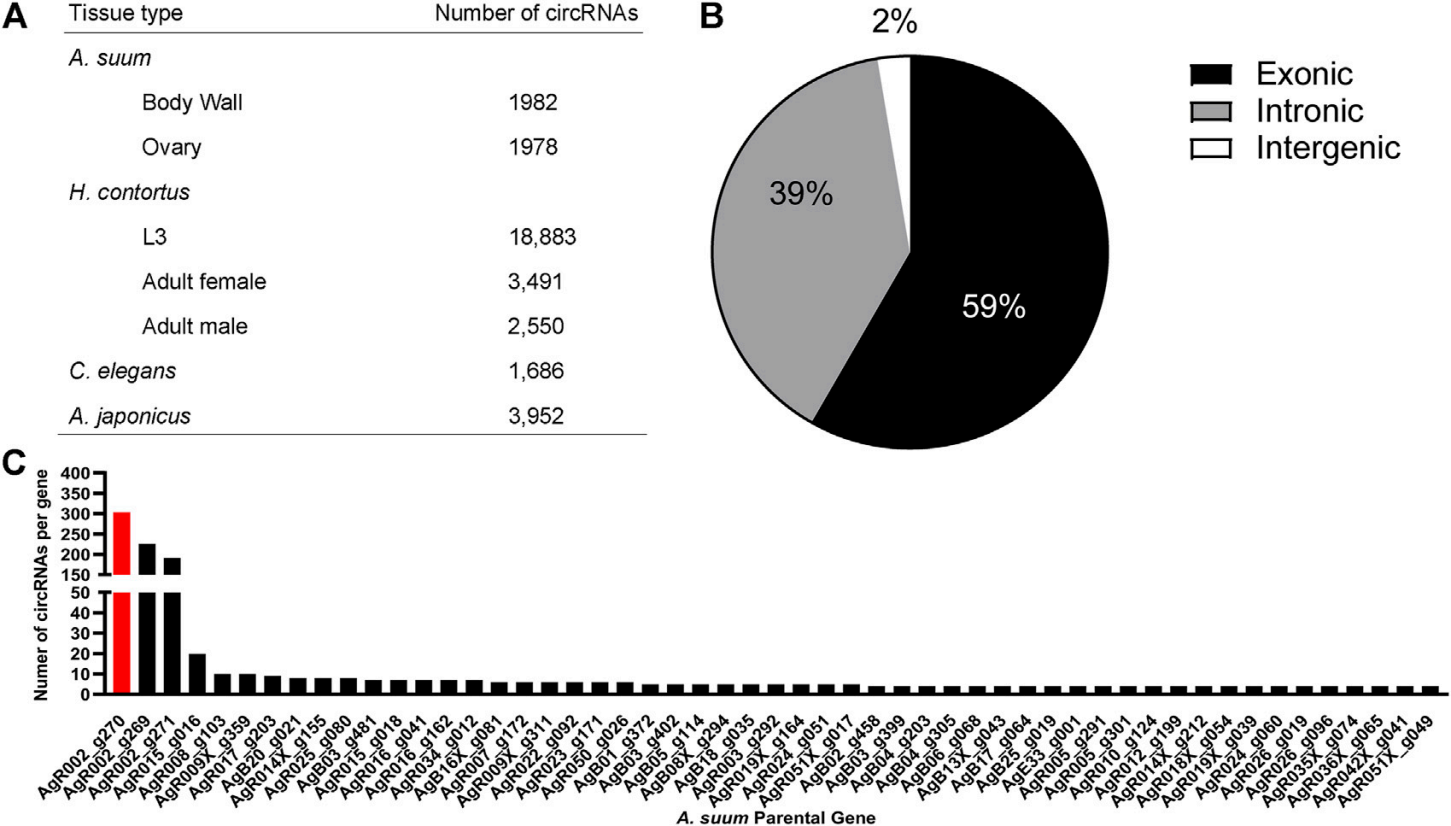
The circRNA complement of *Ascaris suum* is complex and contains exonic, intronic and intergenic circRNAs. **(A)** 1,982 circRNAs were identified in *A. suum* body wall tissue and 1,978 circRNAs were identified in ovary-enriched tissue. The total number of distinct *A. suum* circRNAs identified in both tissues was 1,997. By comparison, 20,073 circRNAs have been identified in the small ruminant gastrointestinal nematode *Haemonchus contortus* (Zhou et al., 2021), with the highest number found in the L3 stage (18,883). In the free-living nematode *Caenorhabditis elegans*, 1,686 exonic circRNAs are known across multiple life-stages (Memczak et al., 2013; Ivanov et al., 2015; Cortés-López et al., 2018), while 3,952 circRNAs have been identified in *Apostichopus japonicus* adults (Zhao et al., 2019). **(B)** The most abundant form of circRNAs found in *A. suum* were exonic circRNAs, followed by intronic circRNAs with the least abundant being intergenic circRNAs. This is consistent with circRNA profiles in other species. **(C)** A frequency distribution showing the number of circRNAs derived from specific *A. suum* genes. Only those genes generated at least four circRNAs are shown. Three *A. suum* genes are particularly enriched, with AgR002_g270 (red) had the highest amount of circRNAs (generating the greatest number of circRNAs, 303).

To examine the presence of circRNA in *A. suum* tissues, a total of six independently prepared samples (three ovary-enriched, three body wall) were used for circRNA sequencing. After the removal of redundant or duplicated circRNAs, we identified 1,982 circRNAs in body wall tissue and 1,978 circRNAs in ovary-enriched tissue, for a total of 1,997 unique and distinct circRNAs (Figure 2A). There were a significant number of circRNAs shared between the two tissue types (1,963) and only 34 circRNAs were identified through circRNA-seq analysis as having tissue-specific expression: 15 circRNAs were identified only in the ovary-enriched samples and 19 were body wall specific. Tissue specific circRNAs are listed in Supplementary Table S1 If *Ascaris* circRNAs are functional, such a general spatial distribution pattern suggests either that the majority of *A. suum* circRNAs are linked to the regulation of transcriptional processes that are broadly conserved across different cell types, or that any functional specificity within tissues is driven by a more restricted temporal or spatial expression of interacting partners rather than the circRNAs themselves. Raw data and circRNA sequences can be viewed using bio-project number PRJNA750737 with SRA numbers, SRR15295818 - SRR15295823.

Of the total 1,997 circRNAs identified in ovary-enriched and body wall tissues, 1,178 (59%) were exonic, 779 (39%) were intronic and 40 (2%) were intergenic (Figure 2B). Whilst there is a lack of data distinguishing the functional relevance of exonic versus intronic circRNAs, there is the potential for exonic circRNAs to be translated into proteins (Legnini et al., 2017), and those translated proteins could have important biological roles.

The number of exons per circRNA was calculated and on average, circRNAs contained approximately three exons with 83% of circRNAs composed of multiple exons (two or more). The number of circRNAs per *A. suum* gene can be viewed in Figure 2C. Interestingly, 37% (752) of circRNAs seem to be derived from chromosome AgR001, while one specific gene locus, AgR002_g270, had the most derived circRNAs, a remarkable 15% of the total (303) (Figure 2C). AgR002_g270 does not have a known or annotated function, or any identified ortholog or paralogs associated with it, but blast analysis (https://blast.ncbi.nlm.nih.gov) of the AgR002_g270 coding sequencing returns ribosomal proteins from various nematode species, including *A. lumbricoides*, *Ascaridia galli*, *Toxocara cati,* and *Baylisascaris procyonics* (U.S. National Library of Medicine, 2022).

### GO and KEGG Analysis of circRNA Parental Genes

**FIGURE 3:**
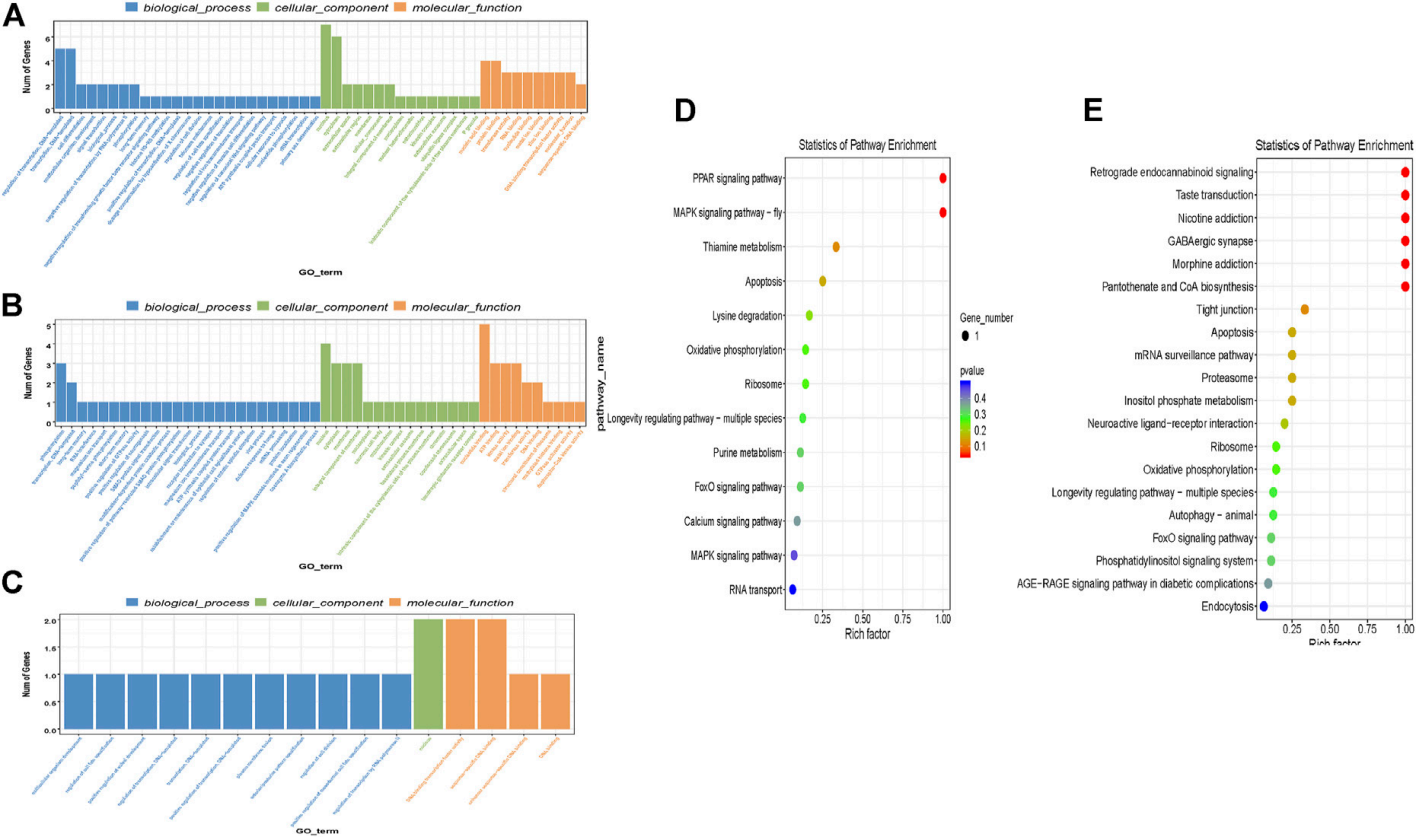
GO and KEGG term analysis of differentially expressed circRNAs identified in *Ascaris suum* ovary-enriched and body wall tissues. The parental genes of circRNAs differentially expressed between *A. suum* ovary-enriched and body wall samples were subjected to GO and KEGG analysis. **(A-C)** CircRNA-seq was performed in biological triplicate and GO analyses of parental genes generating the differentially expressed circRNAs between ovary-enriched and body wall tissues was identified in each sequencing run and are presented here. GO terms are binned according to process (biological process, cellular function, molecular function) and the number of genes in each bin are described on the *y*-axis. **(D,E)** Enriched KEGG terms for parental genes that derived differentially expressed circRNAs identified by comparing ovary-enriched and body wall tissue samples. One of the three sequencing runs did not yield significant differences in this KEGG analysis and is not included. Rich factor (*x*-axis) is the ratio of the number of differentially expressed genes annotated in a pathway. The color and size of each bubble represent *p*-value and the number of genes enriched in a pathway.

CircRNA have the potential to encode proteins (Legnini et al., 2017) so understanding the function of parental genes from which *Ascaris* circRNA derive could provide valuable information about circRNA function. GO and KEGG annotation analyses were conducted to predict possible functions of parental genes (Figure 3). Significant GO and KEGG terms were calculated by hypergeometric equation (Erdélyi et al., 1953) and terms with *p*-values less than 0.05 were defined as significant. Significant GO terms were divided into three groups, biological process, cellular component, and molecular function. 49 GO terms were involved in the biological process category for all three replicates (duplicates were excluded). The most enriched GO terms in the biological process category included “regulation of transcription; DNA templated” (GO:0006355), “transcription, DNA templated” (GO:0006351); and “phosphorylation” (GO:0016310) (Figures 3A–C). For cellular component, 41 individual GO terms were significantly enriched. They mainly consisted of “nucleus” (GO:0005634) and “cytoplasm” (GO:0005737). Molecular function had one enriched GO term, “nucleotide binding” (GO:0000166) (Figures 3A–C).

KEGG pathway analysis was also carried out to determine further significant pathways of circRNA parental genes and identify enriched pathways. There was a total of three different KEGG comparison groups, one for each of the ovary and body wall samples that were submitted from the same adult female worm. Of the three different comparisons that were analyzed for differential expression, one sample comparison did not have any significant differentially expressed KEGG terms and is not included in this analysis. In the two other comparisons, the most enriched KEGG pathways for differential expression between ovary-enriched and body wall tissues include PPAR signaling pathway, MAPK signaling pathway, and retrograde endocannabinoid signaling (Figures 3D,E). 10 of 28 differentially expressed KEGG pathways were involved in “signaling”, suggesting that circRNA parental genes are involved with signaling, signal transduction pathways and other important cellular processes that may support worm viability. If *A. suum* circRNAs are translated and yield functional proteins, this GO and KEGG term analysis points to possible functions that circRNAs could be performing in the worm, based on the parental gene.

### qRT-PCR Tissue Validation of A. suum circRNA Expression in Ovary-Enriched and Body Wall Tissue

**FIGURE 4:**
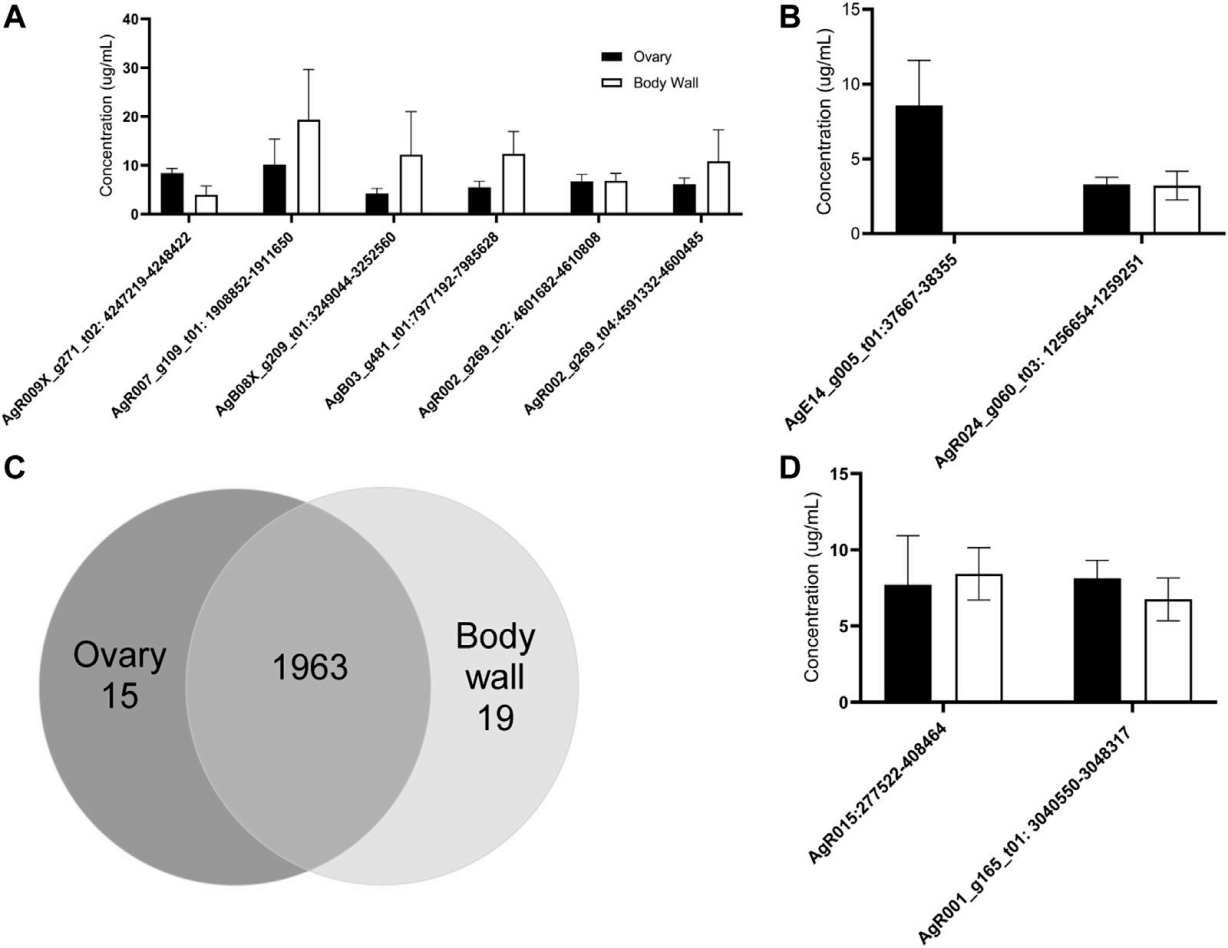
RT-qPCR validates the expression of 10 of individual circRNAs in *Ascaris suum* ovary-enriched and body wall tissues. The tissue expression of circRNAs identified through sequencing was validated using RT-qPCR analysis. C_t_ values were normalized using standard curve analysis with spike in RNA to calculate concentration of each circRNA. N = 4 (minimum), Mean ± SEM, *p* ≤ 0.05 being significant throughout. **(A)** The expression of six prioritized circRNAs was confirmed in ovary-enriched and body wall samples. These circRNAs were selected because of predicted expression in both tissue samples. Expression in both samples was confirmed, with no significant difference in expression levels between tissue samples. **(B)** The spatial expression of two circRNAs with predicted tissue specific distribution in our circRNA sequencing datasets was analyzed. AgE14_g005_t01:37667–38355 was expected to be expressed in ovary-enriched tissue only and this was validated. Although AgR024_g060_t03:1256654–1259251 was expected to be expressed in body wall tissue only based on sequencing data, RT-qPCR analysis suggested a broader spatial distribution pattern (N = 3 minimum). **(C)** circRNA-seq identified 1,963 circRNAs in both tissue types, 15 were found only in the ovary-enriched samples while 19 were specific to the body wall preparations. **(D)** We validated the expression of two atypically large circRNAs (over 5 kb) by RT-qPCR. Amplification using primers spanning back-splice junctions indicates these large RNA molecules are circRNAs.

We used qRT-PCR to confirm and validate the expression of six individual circRNAs identified in *A. suum* samples using circRNA-seq. The six initial circRNAs were selected due their high-count numbers from sequencing data in both ovary and body wall tissues. Divergent primers spanning back-splice junction sites were designed for each circRNA and can be viewed in Supplementary Table S2. Using an RNA exogenous spike in approach allowed us to calculate circRNA concentration levels using a standard curve, and as expected we did not observe any statistical significance in the abundance of individual circRNAs between the two tissue types (N = 5) (Figure 4A). This data validated the circRNA-seq approach as a means to broadly describe circRNA expression but our subsequent qRT-PCR analyses of differentially expressed circRNAs underscored the importance of verifying the circRNA-seq data with secondary methods (Figures 4B,C). The circRNA-seq datasets identified AgE14_g005_t01:37667–38355 as specifically expressed in the ovary-enriched samples and AgR024_g060_t03: 1256654–1259251 in the body wall samples. A full list of tissue specific circRNA expression for both ovary and body wall can be viewed in Supplementary Table S1. Although our qRT-PCR data confirmed that AgE14_g005_t01:37667–38355 was indeed localized to ovary tissue, AgR024_g060_t03: 1256654–1259251 was found to be expressed in both tissue types (Figure 4B). This result could be due to the ability of qRT-PCR to amplify partially degraded transcripts and has been observed in other species (Westholm et al., 2014; Zhao et al., 2019). Alternative explanations could be contamination of the ovary-enriched sample with body wall circRNA during dissection or from cross-contamination of RNA samples. It underscores the need for increased depth of circRNA sequencing married with additional validation measures to confirm spatial localization of circRNAs.

Further validation of select circRNA expression was performed, specifically, of AgR015: 277523–408464 and AgR001_g15_t01: 3040550–3048317. These circRNAs were prioritized due to their large size: AgR015: 277523–408464 was 130,941 nt long and AgR001_g15_t01: 3040550–3048317 was 7,767 nt long. The full length of these circRNAs was calculated from next generation sequencing data. We were able to confirm that these two RNA molecules are circRNAs through qPCR validation (Figure 4D) by creating primers specific to back-splice junction sites. This approach demonstrated both these RNAs form a circular structure and are not spurious background artiffacts or RNA molecules residual from RNase R digestion, even though they are larger in size than typical circRNAs (approximately 500–600 nt) (Ding et al., 2018).

### circRNAs Are Secreted in Extracellular Vesicles, but circRNA Secretion or Tissue Expression is Not Grossly Affected by Ivermectin Treatment

**FIGURE 5:**
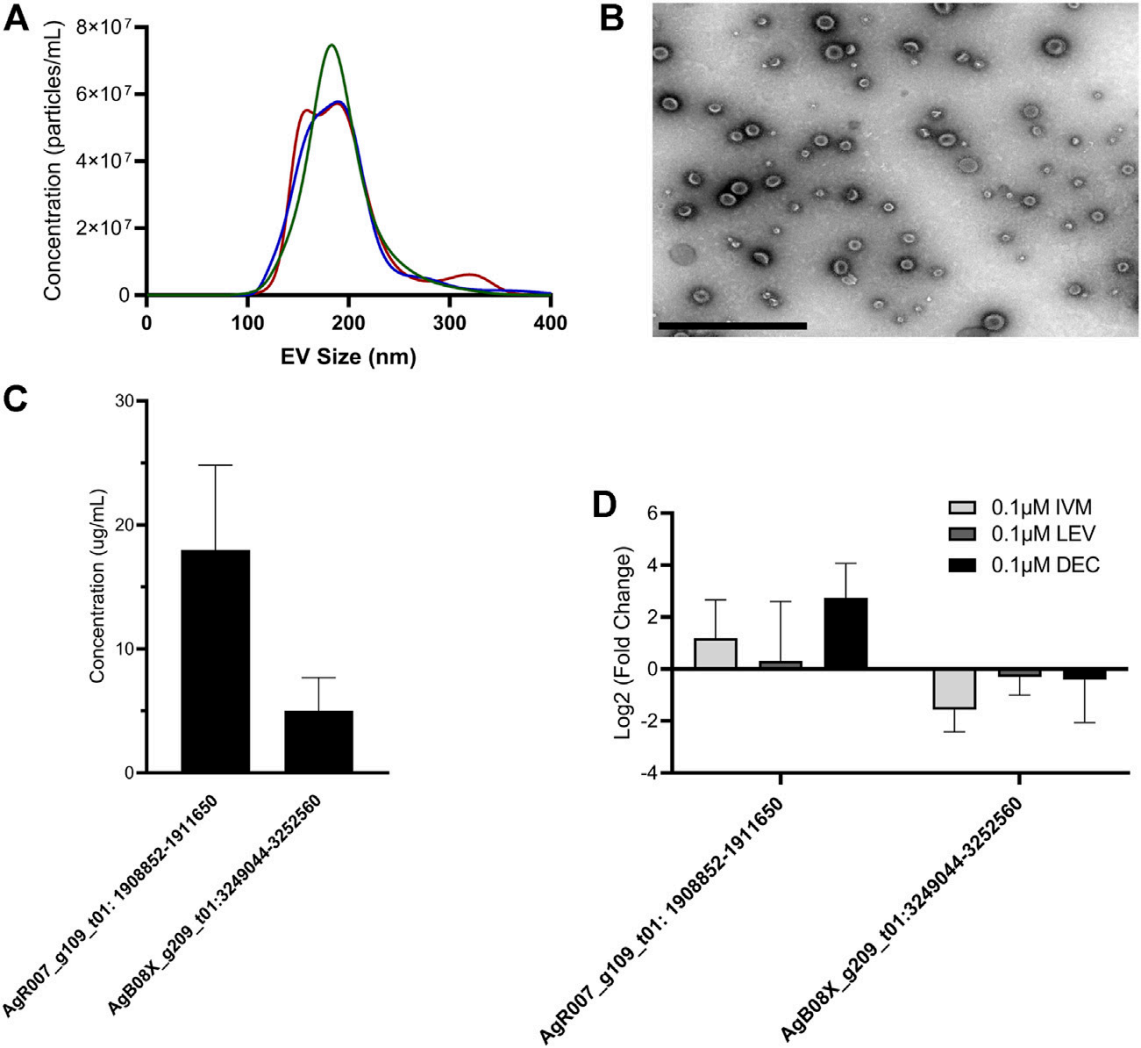
circRNA expression from *Ascaris suum* extracellular vesicles (EVs) is unaffected by anthelminthic drug treatment. **(A)** *Ascaris* EVs were isolated using differential ultracentrifugation and nanoparticle tracking analysis (NTA) was used size and quantify EV population. Mean EV size was 194 nm. Size profile for three independent EV isolations is shown. **(B)** Representative electron micrograph showing *A. suum* EV population. Scale bar 1 µm. **(C)** circRNA expression levels in EVs isolated from untreated *A. suum* was determined using RT-qPCR. Only two of the six circRNAs from Figure 4A were detected in *A. suum* EVs with accurate reproducibility. N = 3 (minimum), Mean ± SEM, *p* ≤ 0.05 **(D)** circRNA expression in EVs was unaffected by 24 h treatment of parental parasites with therapeutically relevant doses of the anthelmintic drugs ivermectin (IVM), diethylcarbamazine (DEC), or levamisole (LEV). circRNA expression in EVs was normalized to EVs secreted by untreated control (N = 3 minimum, Mean ± SEM, *p* ≤ 0.05).

circRNAs have been found to be secreted from mammalian parental cells in extracellular vesicles (EVs) (Lasda et al., 2016). Many species of nematodes are known to secrete EVs (Buck et al., 2014; Zamanian et al., 2015; Tzelos et al., 2016; Tritten et al., 2017; Eichenberger et al., 2018a; Eichenberger et al., 2018b; Harischandra et al., 2018; Shears et al., 2018; Hansen et al., 2019) but the presence of circRNAs in those vesicles has not been explored. We hypothesized that *A. suum* EVs would contain circRNAs. To test this hypothesis, we used qRT-PCR to quantify the abundance of select circRNA transcripts in *A. suum* EVs using the spike-in approach as previously described in section 3.3. We first isolated EVs from conditioned media, imaged the vesicles using TEM and performed nanoparticle tracking analysis (NTA) on the isolated samples to confirm EV morphology, size profile and concentration (Figures 5A,B). We tested these EVs for the presence of the same six circRNAs that were also tested for in tissue qRT-PCR validation (Figure 4A). Of these, two circRNAs (AgR007_g109_t01: 1908852–1911650 and AgB08X_g209_t01:3249044–3252560) could be consistently amplified from EV RNA samples (N = 3) (Figure 5C). These data indicate that parasitic nematode circRNAs are secreted into the host milieu in EVs. We also looked for the presence of circRNAs in *A. suum* supernatants generated through EV isolation, representing non-EV mediated mechanisms of circRNA secretion. This approach yielded insufficient amounts of total RNA to conduct RT-qPCR analysis suggesting EVs may represent the primary mechanism of circRNA secretion from this worm.

Given previous data published by our laboratory on the inhibitory effect of ivermectin (IVM) on parasitic nematode EV secretion, we examined whether IVM would inhibit circRNA secretion via EVs. Parasites were cultured in the presence or absence of 0.1 µM (Figure 5D) or 1 µM IVM (Supplementary Figure S1) to model a therapeutically relevant dose. After 24 h, parasite media was collected, total EV RNA extracted and used in RT-qPCR. When worms were treated with 0.1 µM or 1 µM IVM we did not observe any decrease in AgR007_g109_t01: 1908852-1911650 or AgB08X_g209_t01:3249044-3252560 abundance in isolated EVs (Figure 5D, Supplemental Figure 3, N = 3). This observation was perhaps surprising, given the strong and consistent evidence for an inhibitory effect of IVM on EV secretion in parasitic nematodes, including *Ascaris* (Harischandra et al., 2018; Loghry et al., 2020). This may point to other non-EV mediated routes of circRNA release from these worms. Other anthelmintic drugs are reported to have sporadic inhibitory effects on EV secretion by some life stages of filarial parasitic nematodes (Loghry et al., 2020). Therefore, we also looked at the effect of diethylcarbamazine (DEC) and levamisole (LEV) treatment on circRNA expression in *A. suum* EVs (Figure 5D, Supplementary Figure S1). Consistent with the IVM data, we did not see any inhibition in EV circRNA abundance, collectively indicating inhibition of circRNA secretion via EVs is not clearly associated with the mode of action of anthelmintic drugs.

**FIGURE 6:**
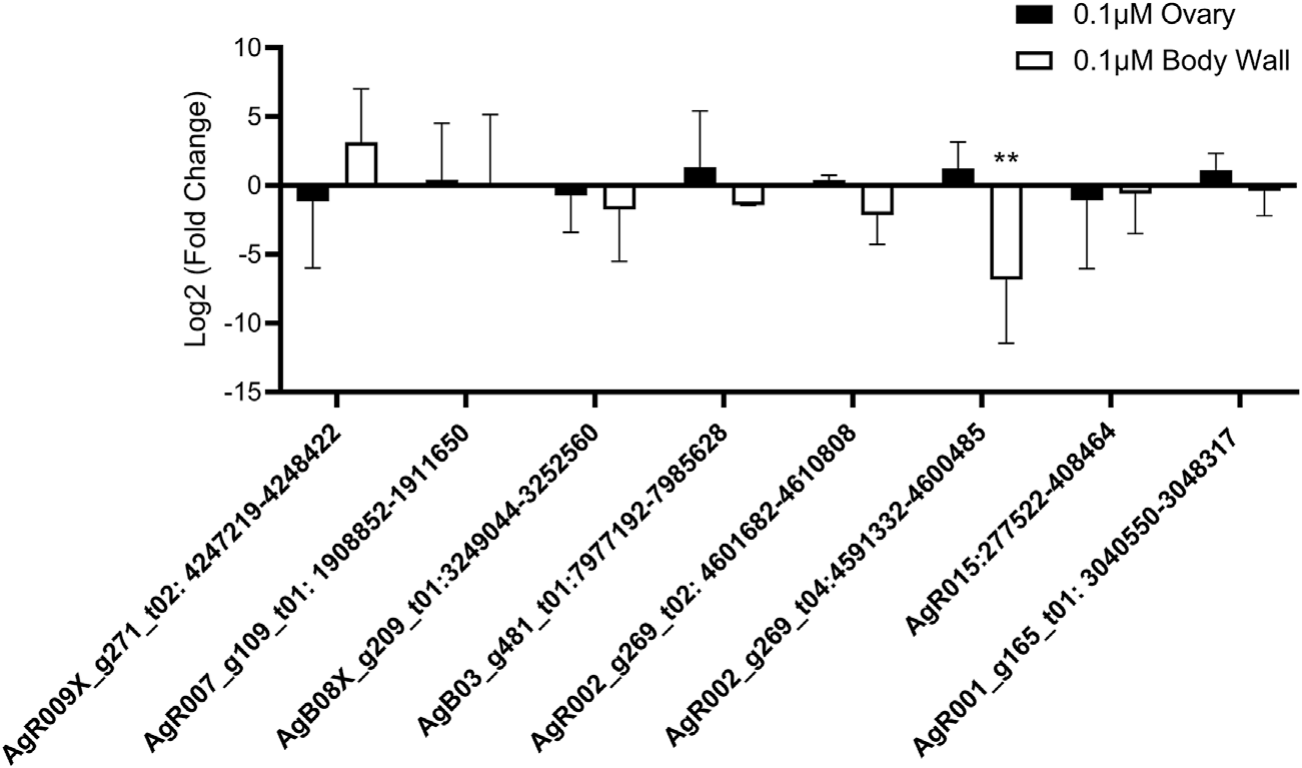
Ivermectin (IVM) treatment has no global effect on circRNA expression in *Ascaris suum* tissues. Individual adult female *A. suum* parasites were treated with IVM for 24 h in culture before ovary-enriched and body wall tissues were extracted for circRNA expression analysis using qRT-PCR. C_t_ values were normalized to 40 ng exogenous spike in RNA using 2^−ΔΔCq^. N = 4 (minimum), mean ± SEM, *p* ≤ 0.05 considered significant (**p ≤ 0.01).

To fully evaluate the effect of anthelmintic drug treatment on circRNA expression, we lastly examined whether IVM, DEC, or LEV had any effect on circRNA expression in *Ascaris* tissues using the same cohort of six prioritized circRNAs. Treatment of worms with 0.1 µM (Figure 6) or 1 µM IVM (Supplementary Figure S2) did not alter expression of any tested circRNA in ovary tissues (N = 4). Similarly, five of the six circRNAs in body wall tissue were unaffected by IVM treatment although AgR002_g269_t04:4591332–4600485 was downregulated by 95% compared to control (*p* = 0.0071, N = 4) in body wall tissue at 0.1 µM (Figure 6), but not at 1 µM (Supplementary Figure S2). Consistent with the results from IVM treated tissues, we did not observe any effect of DEC or LEV (Supplementary Figure S3) on circRNA expression in *A. suum* ovary or body tissues. Collectively our data do not support the hypothesis that anthelmintic drug mechanism of action involves a direct impingement of normal circRNA expression or secretion.

### Ascaris circRNAs are Predicted to act as miRNA Sponges

A well-established functional role for circRNAs is to regulate gene expression by binding miRNAs, effectively acting as miRNA sponges. circRNAs can contain numerous binding sites for individual or multiple miRNAs (Capel et al., 1993; Li F. et al., 2015; Zheng et al., 2016) For instance, murine CDR1as (ciRs-7) has 63 conserved binding sites for the miRNA mir-7 (Hansen T.B. et al., 2013), while circHIPK3 can sponge nine different human miRNAs (Zheng et al., 2016). Here, we wanted to probe potential miRNA interactions with the *A. suum* circRNA dataset to support the hypothesis that *Ascaris* circRNAs can act as miRNA sponges. We used the miRanda algorithm (Enright et al., 2003) to predict interactions between *Ascaris* circRNAs and endogenous *A. suum* miRNAs.

**FIGURE 7:**
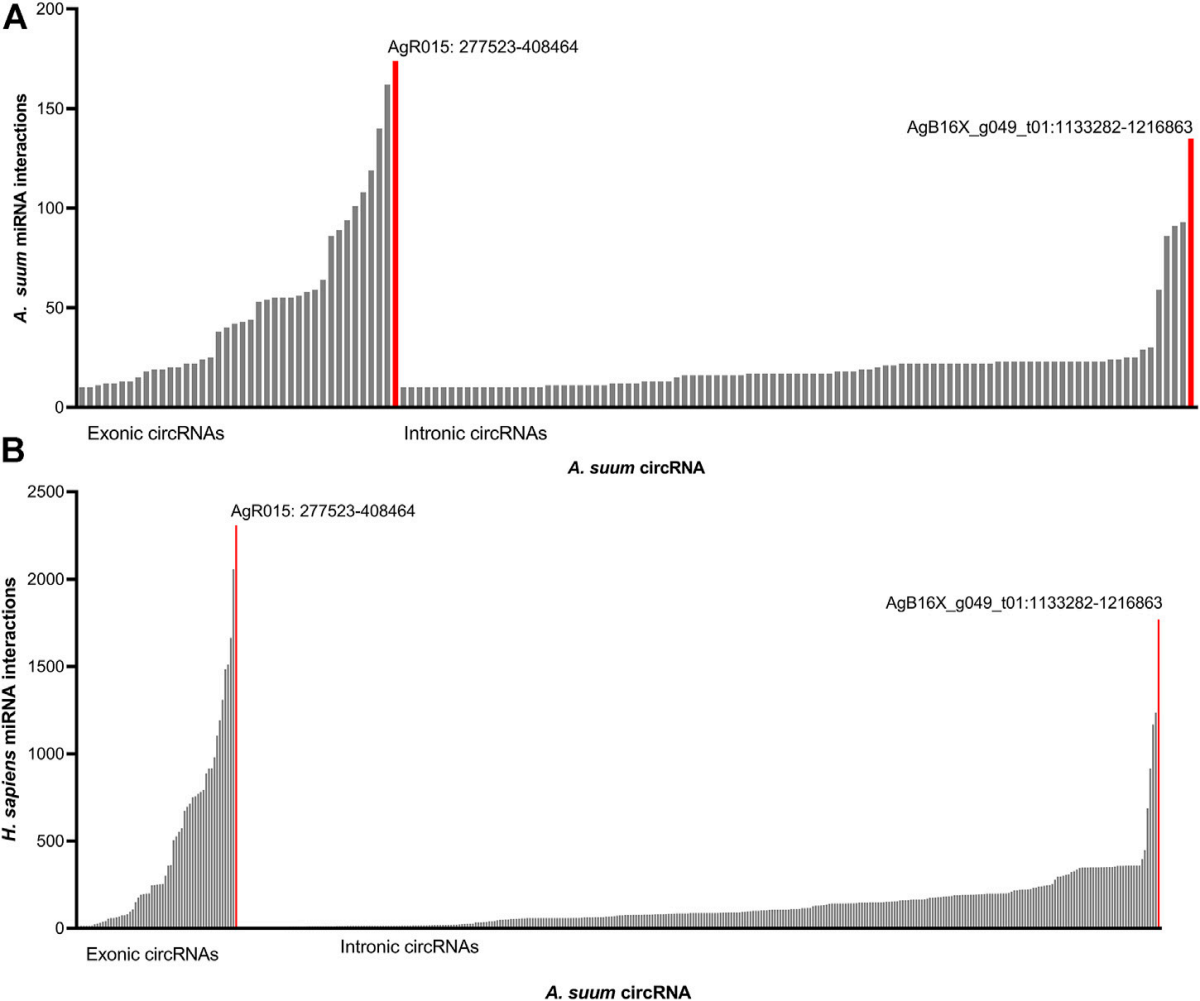
*Ascaris suum* circRNAs interact with both endogenous and host miRNAs. miRanda was used to predict interactions between *A. suum* circRNAs and both endogenous *A. suum* miRNAs as well as host (human) miRNAs. **(A)** Predicted interactions between *A. suum* circRNAs and *A. suum* miRNAs. The number of interactions per circRNA are shown and circRNAs with under 10 miRNA interactions were excluded from these graphs. Exonic circRNAs typically had a more miRNA interactions per circRNA than intronic circRNA. **(B)** Predicted interactions between *A. suum* circRNAs and human miRNAs. The number of interactions per circRNA are shown and circRNAs with under 10 miRNA interactions were excluded from these graphs. Again, exonic circRNAs typically had a more miRNA interactions per circRNA than intronic circRNAs.

Approximately 10% of *A. suum* circRNAs (202 out of 1,997) were predicted to interact with *A. suum* miRNAs (Figure 7A). The number of miRNA interactions per circRNA varied, with AgR015:277522-408464 found to have highest number of distinct miRNA interactions 174) at 19 discrete binding sites on the circRNA molecule, illustrating that different miRNAs can bind to the same sites on circRNAs. Interestingly, for both human and *Ascaris* miRNAs, exonic circRNAs exhibited a higher number of predicted miRNA interactions than intronic circRNAs (Figure 7). The highest number of interactions between exonic circRNAs and *Ascaris* miRNAs was 174 (AgR015: 277523-408464), while 135 (AgB16X_g049_t01:1133282-1216863) interactions was observed to be the highest for intronic circRNAs. Interactions between exonic and intronic circRNAs with *Ascaris* and host miRNAs is summarized in Table 1.

**TABLE 1:** Summary of predicted circRNA-miRNA binding by circRNA type. Exonic circRNAs were observed to have the highest number of predicted interactions for both human and *Ascaris* miRNAs. Human miRNAs also were observed to have a significantly higher number of interactions for each of the two types of circRNAs as compared to worm miRNAs. The significant differences in exonic and intronic circRNA expression has not yet been fully established, but could be due to the ability of exonic circRNAs being translated.

circRNA Type	Highest number of interactions	Average Number of interactions
Exonic circRNA		
*A. suum* miRNAs	174	32
*H. sapiens* miRNAs	2,308	281
Intronic circRNA		
*A. suum* miRNAs	135	6
*H. sapiens*	1,769	113

**FIGURE 8:**
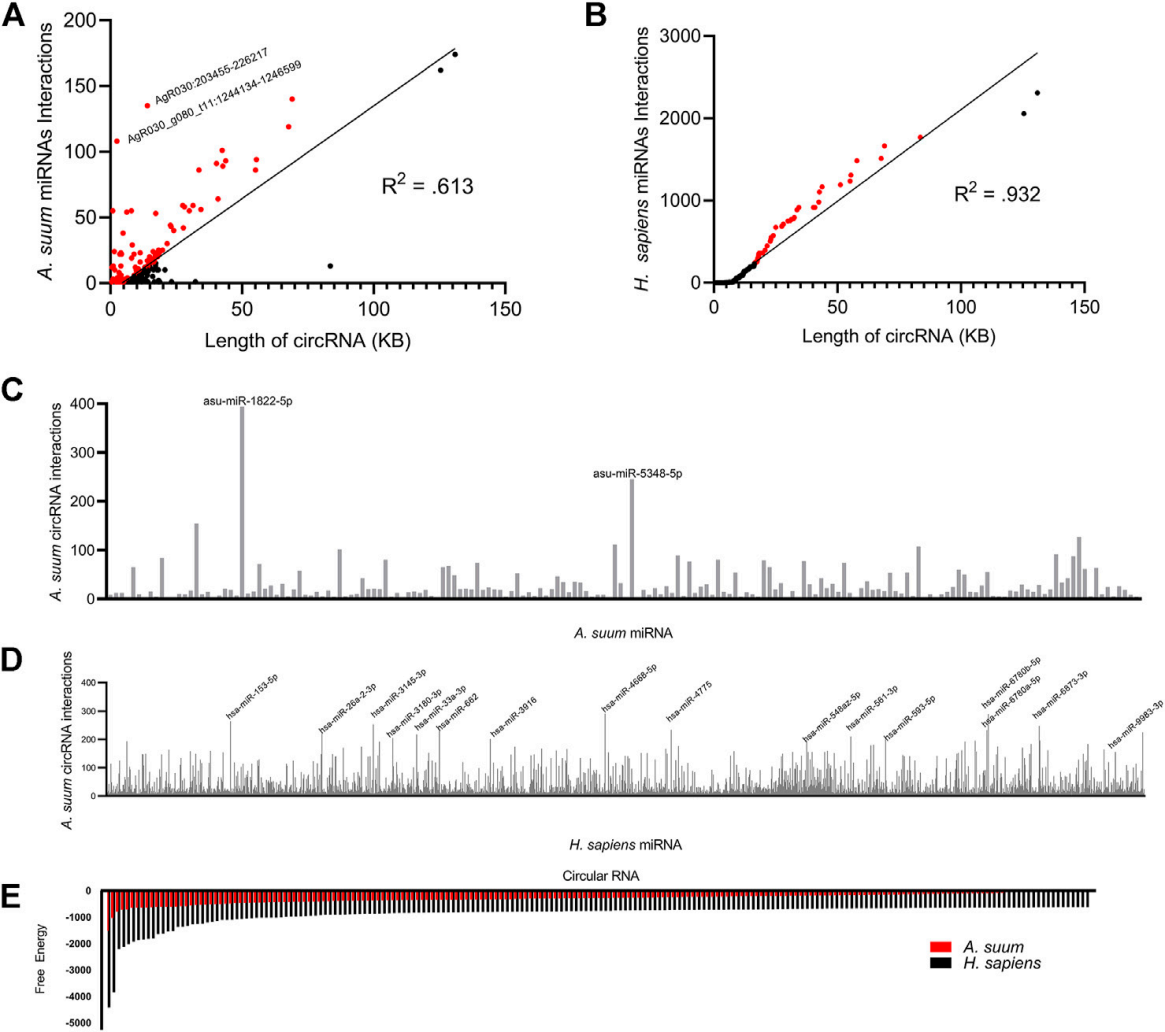
A profile of *Ascaris suum* circRNA-miRNA interactions. miRanda was used to predict *Ascaris* circRNA interactions with both endogenous *Ascaris* miRNAs and human host miRNAs. The number of *Ascaris* miRNA interactions **(A)** and human miRNA interactions **(B)** was plotted against individual circRNA length using a line of best fit. A less strong correlation between circRNA length and *Ascaris* miRNA interaction suggests *Ascaris* circRNAs are enriched for *Ascaris* miRNA binding sites. CircRNAs enriched for miRNA binding sites relative to their length are highlighted in red. The frequency of binding events for individual *Ascaris* **(C)** and human **(D)** miRNAs at *Ascaris* circRNAs was plotted. Only miRNAs with 10 or more interactions were included. Highlighted individual miRNAs had over 200 interactions with *Ascaris* circRNAs. **(E)** The miRanda free energy score was used to assess *Ascaris* circRNA-miRNA binding strength. Lower miRanda free energy scores are associated with a more thermodynamically stable bond between circRNA and miRNA. miRanda scores suggest *Ascaris* cirRNA-*Ascaris* miRNA interactions (red) are weaker than *Ascaris* cirRNA-human miRNA interactions. Only the lowest 200 free energy scores are included.

To determine if the number of miRNA interactions per circRNA was simply a reflection of circRNA length, we normalized the miRNA interaction number to length of each circRNA (Figure 8A). There was not a strong linear correlation between length of *A. suum* circRNA length and *A. suum* miRNA interaction number (*R* ^2^ = 0.613), suggesting that the number of *A. suum* miRNAs with which an *A. suum* circRNA interacts is not strongly correlated with the length of circRNAs and by extension, that some *A. suum* circRNAs are explicitly enriched in miRNA interaction sites. For example, AgR030:203455–226217 (14 KB) and AgR030_g080_t11: 1244134–1246599 (2 KB) are both enriched by a high number of miRNA interactions (Figure 8A).

In addition to examining the number of miRNA interactions for each *A. suum* circRNA, we also wanted to probe the interaction from the opposite direction and determine if any *A. suum* were specifically enriched in binding to *A. suum* circRNAs. In total, we observed that 180 distinct *A. suum* miRNAs were predicted to interact with *A. suum* circRNAs (Figure 8C). Two worm miRNAs had over 200 circRNA interactions, asu-miR-1822-5p and asu-miR-5348-5p (Figure 8C), but there are no known functions or phenotypes associated with these two miRNAs so postulating some functional relevance to this miRNA sponging activity is not possible. asu-miR-1822-5p did have the highest amount of circRNA interactions of all of *Ascaris* miRNAs at 394 total predicted interactions.

The secretion of *A. suum* circRNAs into the host environment in EVs and the potential for delivery of those circRNAs to host tissues seeded the possibility that secreted circRNAs could be acting as sponges for host (human) miRNAs. Therefore, we also used miRanda to predict interactions between *Ascaris* circRNAs and host (human) miRNAs. There were more predicted interactions between *Ascaris* circRNAs and human miRNAs, with a total of 398 distinct circRNAs interacting with host miRNAs (Figure 7B). AgR015:277522–408464 was predicted to have the highest number of miRNA interactions (2,308). This disparity may reflect the greater number of annotated miRNAs in the human genome compared to that of *Ascaris.* AgR015: 277523–408464 (exonic) and AgB16X_g049_t01:1133282–1216863 (intronic) were predicted to have the highest number of interactions across all miRNA-circRNA interactions, suggesting that these circRNAs contain a high number of binding sites. There is no supporting data in the circRNA literature, however, to support a functional difference between miRNA sponging by exonic versus intronic circRNAs.

There was a total of 577 circRNAs that were predicted to interact with *A. suum* miRNAs and 645 circRNAs that were predicted to interact with human miRNAs. While the total number of circRNA interactions for both *A. suum* and human miRNA were similar, the number of *A. suum* miRNAs interactions per individual circRNAs was almost 10 times fewer than the number of human miRNA interactions per circRNA (Figure 7), suggesting that *A. suum* circRNAs could have greater binding affinity for human miRNAs. 79% of circRNA–*A. suum* miRNA interactions had under 10 interactions per circRNA, while only 41% of circRNA–human miRNA interactions were under 10 interactions per circRNA. This suggests that while there is not a large difference in the number of parasite or human miRNAs that are predicted to bind *Ascaris* circRNAs, the number of miRNAs sponging to each circRNA is discrepant and *A. suum* circRNAs tend to have more binding sites for human miRNAs than *A. suum* miRNAs.

To determine if host miRNA binding was correlated with circRNA length, we also normalized circRNA length to the number of predicted host miRNA interactions (Figure 8B). In contrast to endogenous *Ascaris* circRNA-*Ascaris* miRNA interactions, there does seem to be a stronger linear correlation in this relationship (*R* ^2^: 0.932), suggesting that there is no specific enrichment of human miRNA binding to *A. suum* circRNAs.

Again, we observed a larger number of circRNA interactions occurring with human miRNAs as compared to *A. suum* miRNAs. Human miRNAs had a total of 2,414 predicted circRNA interactions with 16 miRNAs having over 200 circRNA interactions (Figure 8D). Relative to the *Ascaris* miRNAs that were predicted to frequently bind to *Ascaris* circRNAs, more functional information is known about these human miRNAs that are predicted to bind to *Ascaris* circRNAs, among them hsa-miR-4668-5p, which had the highest number of predicted circRNA interactions at 291. hsa-miR-4668-5p has been implicated in regulating TGF-beta signaling (Bhardwaj et al., 2020). TGF-beta is a cytokine involved in the proliferation, differentiation and function of lymphocytes, macrophages, and dendritic cells (Kubiczkova et al., 2012) and dysregulation of TGF-beta through circRNA sponging could potentially be another strategy these parasitic worms use to manipulate and modulate the host immune response. hsa-miR-6780b-5p had the second highest number of predicted circRNA interactions at 289. This miRNA has been linked to insulin resistance in hepatocellular carcinoma cells (HepG2) (Li et al., 2020) and whilst not directly related to immune system function, there could be other processes that could be altered as a result of its sequestering by circRNA sponging that could be advantageous to parasite infection. The recognition that a human immunomodulatory miRNA, hsa-miR-4668-5p, strongly interacts with secreted parasite circRNAs prompted us to examine whether any of the other human miRNAs that were predicted to bind to *Ascaris* circRNAs had known immunoregulatory functions. The percentage of miRNAs with documented immunoregulatory roles as a function of the total number of miRNAs predicted to bind parasite circRNAs was calculated (Table 2). Although this did not present a strong argument that immunomodulatory miRNAs are explicitly enriched for parasite circRNA binding, some interesting miRNAs were noted, including hsa-let-7, which had the highest percentage (0.5461) and is known to influence T-cell activation and mediates cytokine expression (Gilles and Slack, 2018).

**TABLE 2:** Parasite circRNAs are predicted to interact with host miRNAs that have immunoregulatory functions. Frequency of each miRNA was calculated by counting the number of individual miRNAs in the sample. There was a subset of host miRNAs with predicted interactions to A. suum circRNAs that are associated with immunomodulatory functions. While known immunomodulatory miRNAs did not take up a large sum of the host miRNA demographic, miRNA with functions not directly associated with the immune system could still be affecting the host-parasite immune interface and carry beneficial functions to parasite infection.

miRNA	Function	Frequency of miRNAs in data
hsa-let-7a-2-3p, hsa-let-7a-3p, hsa-let-7a-5p, hsa-let-7b-3p, hsa-let-7b-5p, hsa-let-7c-3p, hsa-let-7d-3p, hsa-let-7d-5p, hsa-let-7e-3p, hsa-let-7f-1-3p, hsa-let-7f-2-3p, hsa-let-7f-5p, hsa-let-7g-3p, hsa-let-7i-3p, hsa-let-7i-5p	Reduce IL-6 expression (Chandan et al., 2016)	0.546172705
hsa-miR-10a-3p, hsa-miR-10a-5p, hsa-miR-10b-3p, hsa-miR-10b-5p	T-reg cell differentiation from CD4+ T-cells, decrease mucosal inflammatory response and inhibit Th1 and Th17 cell function, inhibit NF-kB activation (Tahamtan et al., 2018)	0.078997548
hsa-miR-124-3p, hsa-miR-124-5p	Induces anti-inflammatory effects through downregulation of TLR-6 and Myd88 (Qin et al., 2016)	0.032688641
hsa-miR-126-3p, hsa-miR-126-5p	Higher expression in response to anti-atherogenic triglyceride-rice lipoproteins or polyunsaturated fatty acids treatment. (Chandan et al., 2016)	0.039498774
hsa-miR-132-3p, hsa-miR-132-5p	Suppresses NF-kB nuclear translocation and the production of STAT3 (Tahamtan et al., 2018)	0.036774721
hsa-miR-145-3p, hsa-miR-145-5p	Increase release of TNF-alpha (Chandan et al., 2016)	0.110324162
hsa-miR-146a-3p, hsa-miR-146a-5p, hsa-miR-146b-3p, hsa-miR-146b-5p	Upregulation of IL-1 and inhibit inflammatory response (Hirschberger et al., 2018)	0.265595206
hsa-miR-150-3p, hsa-miR-150-5p	Regulates genes whose downstream products encourage differentiating stem cells towards becoming megakaryocytes and involved in controlling B and T cell differentiation (Lu et al., 2008)	0.01225824
hsa-miR-155-3p, hsa-miR-155-5p	Regulates DC maturation (Chandan et al., 2016)	0.044946881
hsa-miR-181a-2-3p, hsa-miR-181a-3p, hsa-miR-181a-5p, hsa-miR-181b-2-3p, hsa-miR-181b-3p, hsa-miR-181b-5p, hsa-miR-181c-5p, hsa-miR-181d-3p, hsa-miR-181d-5p	Enhancement of TCR signaling and phosphorylation of immunoreceptor, increased M2 polarization (Hirschberger et al., 2018)	0.603377826
hsa-miR-187-3p, hsa-miR-187-5p	Regulates cytokine production (Chandan et al., 2016)	0.017706347
hsa-miR-21-3p, hsa-miR-21-5p	Plays an essential role in the negative feedback pathway of inflammation (Tahamtan et al., 2018)	0.062653228
hsa-miR-221-3p, hsa-miR-221-5p	Downregulates TNF-alpha (Chandan et al., 2016)	0.0204304
hsa-miR-222-3p, hsa-miR-222-5p	Decrease ICAM-1 expression and restricts interactions of cytotoxic T lymphocytes (Chandan et al., 2016)	0.019068374
hsa-miR-223-3p, hsa-miR-223-5p	Decreases accumulation of NLRP3 and inhibits IL-1b production from the inflammasome (Hirschberger et al., 2018)	0.102152002
hsa-miR-24-1-5p, hsa-miR-24-2-5p, hsa-miR-24-3p	Increases the production of Arg1, CCL17, CCL-22, CD163, and CD206 in unstimulated macrophages (Chandan et al., 2016)	0.040860801
hsa-miR-29a-3p, hsa-miR-29c-5p, hsa-miR-29c-3p, hsa-miR-29b-3p, hsa-miR-29b-2-5p, hsa-miR-29b-1-5p, hsa-miR-29a-5p	Increases apoptosis in cells with overexpression (Liston et al., 2012)	0.476709344
hsa-miR-34a-3p, hsa-miR-34a-5p	Biomarker for hepatitis-related hepatocellular carcinoma (Hirschberger et al., 2018)	0.133478616

To determine if circRNA could form a stable bond to worm and human miRNAs, we examined at the miRanda free energy score. A lower miRanda free energy score is associated with a more thermodynamically stable bond, and therefore, a stronger bond between circRNA and miRNA. We found that circRNAs form a more stable bond with human miRNAs as compared to *Ascaris* miRNAs based on these free-energy scores (Figure 8E). The free energy scores associated with human miRNAs are almost two times lower than *Ascaris* miRNA free energy scores suggesting the secreted parasite circRNAs could be forming tight bonds to host miRNAs and this could lead to changes in host gene expression through secretion of *A. suum* circRNAs in EVs.

## Discussion

While circRNAs have been described in the free-living nematode *Caenorhabditis elegans* (Memczak et al., 2013; Ivanov et al., 2015) and now a more thorough description from *Haemonchus contortus* (Zhou et al., 2021), a parasitic nematode of small ruminants, our understanding of circRNA expression and function in nematodes is lacking. A summary of circRNA biogenesis is presented in Figure 1 and the identification of exonic, intergenic and intronic circRNAs in this study, and others, supports this model in nematodes.

A comparison of circRNAs in *C. elegans* with that of the two parasitic nematodes studied to date points to an overall conservation of circRNA profile. circRNA descriptions in *C. elegans* have focused on exonic circRNAs rather than intergenic or intronic circRNAs, perhaps due to the possibility of protein translation from these exonic circRNAs. A total of 1,686 exonic circRNAs have been identified in various *C. elegans* life stages (Ivanov et al., 2015; Memczak et al., 2013; Cortés-López et al., 2018) (Figure 2A). In comparison, we found a similar number of exonic circRNAs (1,178) in adult female *Ascaris suum* whilst 14,251 exonic circRNA were discovered in *H. contortus* (Zhou et al., 2021) across three life stages (the infective third stage larvae, adult male and adult female worms) (Figure 2A). The number of exonic circRNAs is comparable between *Ascaris* and *C. elegans* but significantly higher in *Haemonchus*, with the greatest number being expressed in the third stage larvae of that species. It is possible that defining the larval circRNA complement in *Ascaris* will reveal a similar level of exonic circRNA complexity. Cortés-López et al. (2018) reported that 98.2% of *C. elegans* circRNAs contained a coding sequence while 1.8% were labeled as “other”. While not explicitly stated in that manuscript, these circRNAs might be considered intronic. Compared to *A. suum* (2%) and *H. contortus* (6%), the percentage of *C. elegans* intronic circRNAs is very similar. GO terms of circRNA parental genes were only assigned for adult *C. elegans* worms with enriched pathways including organism development, determination of adult lifespan, enzyme binding and intracellular components. These GO terms are different from both *H. contortus* and *A. suum* with assigned GO terms in those species more focused on signaling and transcriptional pathways. This suggests that whilst overall circRNA profiles may be conserved between *C. elegans* and parasitic species, that is to say, abundance of exonic circRNAs relative to intronic, the functionality of those circRNAs may be very different.

Comparing the two parasitic nematode species studied to date reveals similarities in their circRNA complement. 71% of *H. contortus* circRNAs are exonic, 22% are intergenic, and 6% are intronic (Zhou et al., 2021). We identified a similar pattern in *A. suum* circRNAs, with the majority coming from exonic regions (59%), followed by intergenic (39%) and the least amount of circRNAs originating from intronic regions (2%). The majority of circRNAs in these two species originate from protein coding regions, and may hint that an important circRNA function could be protein translation. *H. contortus* circRNA parental genes were assigned GO terms such as signaling, signal transduction, protein binding, and receptor activity. Significant GO terms assigned to *A. suum* parental genes included transcription and nucleotide binding, and whilst distinct from the *Haemonchus* assignments, still suggest that circRNAs from both worms could be functioning in important regulatory processes. *H. contortus* KEGG pathways included MAPK signaling among others. Similarly, in the identified *Ascaris* KEGG pathways, MAPK signaling was also enriched. This suggests that some degree of conservation in the function of circRNA parental genes and if proteins are translated from these circRNAs, they could be performing similar functions. The idea that circRNAs derived from exonic linear RNA regions encode functional proteins is well accepted. In *Drosophila,* circRNAs are known to have specific association with translating ribosomes and proteins are generated from circRNA minigenes. circRNAs also contain specific stop codons, supporting endogenous circRNA translation in *Drosophila* fly heads (Pamudurti et al., 2017). In mice, the exonic circRNA circ-ZNF609 contains a reading frame with both a start and stop codon and is associated with polysomes (Legnini et al., 2017). This circRNA is translated into a protein in a cap-independent manner, since circRNAs do not contain a 5’ cap. circ-ZNF609 was transfected into HeLa and N2A cells with two different protein isoforms produced and detected via western blot. circ-ZNF609 is associated with muscular dystrophy in mice and humans and has been shown to regulate myoblast proliferation (Legnini et al., 2017). Clearly there is a precedent for exonic circRNAs to serve as substrates for protein translation and this may be an important function in parasitic nematodes.

One hallmark function of circRNAs is that of a miRNA sponge. circRNAs have the ability to alter gene expression by binding miRNAs, reducing their bioavailability and leading to their loss of function. This process has been established in many organisms including humans (Panda, 2018), mice (Hansen et al., 2013), and *Drosophila* (Westholm et al., 2014). In humans, circRNA-miRNA sponging has been extensively studied within the context of human disease. CDR1as, the first circRNA-miRNA sponge, was discovered in mice (Hansen T. B. et al., 2013) but has also been identified in other animals, including humans where it binds miR-7 and contains over 60 binding sites for this miRNA. CDR1as has been linked to several diseases such as Alzheimer’s disease (Lukiw, 2013) and hepatocellular carcinoma (Yu et al., 2016) due to this sponging of miR-7. It is possible that parasitic nematode circRNAs could also function as miRNA sponges to regulate key processes in these organisms. 205 of the 1,997 circRNAs identified in adult female *A. suum* were predicted to bind to *A. suum* miRNAs. After normalizing length of circRNA to number of miRNA bindings sites, we found that *A. suum* circRNAs appeared enriched for *Ascaris* miRNA binding sites relative to their length, which may be expected if this is their function. Comparing our *Ascaris* data to that generated from *Haemonchus*, fewer *Haemonchus* miRNAs were predicted to interact with circRNAs 194) across all three life *H. contortus* stages examined. In the sea cucumber, *A. japonicus*, the opposite was observed with 3,679 out of 3,952 circRNAs identified predicted to interact with miRNAs (Zhao et al., 2019). Whilst these variations in predicted circRNA-miRNA interaction could be founded in differences if approach, life stage or tissue types examined, it may also reflect differences in the functional roles for circRNAs across diverse invertebrate species.

Our laboratory and others have shown that miRNAs and other small RNA species are secreted by parasitic nematodes into the host environment via extracellular vesicles (EVs) (Zamanian et al., 2015; Buck et al., 2014; Hansen E. et al., 2019; Gu et al., 2017). Delivery of those EVs to host cells elicits transcriptional changes that benefit the parasite, establishing a mechanism by which parasites can modulate host responses at the genetic level. EVs secreted by the murine gastrointestinal nematode *Heligmosomoides polygyrus* inhibit genes involved in toll-like receptor signaling and IL-33 signaling (Buck et al., 2014), and also suppress macrophage activation through the IL-33 pathway, as well as driving other functionally important response pathways in those cells (Coakley et al., 2017). Filarial nematode parasites also secrete EVs that modulate macrophage phenotypes (Zamanian et al., 2015) and contain a complex miRNA cargo with explicit sequence homology to host miRNAs, suggesting parasite miRNAs could act as host miRNA mimics to affect gene expression. Relevant to this study, miRNAs found encapsulated in *A. suum* EVs are predicted to target important immune response cytokines such as IL- 13, 25, and 33 (Hansen et al., 2019). Here, we identified *Ascaris* circRNAs in EV-enriched fractions of spent culture media, suggesting circRNAs are part of the diverse EV cargo. Further, we found in this study that *Ascaris* circRNAs are predicted to strongly interact with human miRNAs. We posit that parasite circRNAs could be delivered to host cells via EVs and contribute to the transcriptional changes observed at the host-parasite interface. Supporting this hypothesis, secreted circRNAs have been shown to be functionally relevant in a wide variety of pathological settings. In colorectal cancer, circRNAs secreted via EVs have been shown to lead to drug resistance (Wang et al., 2020). EVs containing ciRS-122 from oxaliplatin resistant colorectal cancer cells, were delivered to drug sensitive cancer cells, which then led to resistance to oxaliplatin through sponging of miRNA-122. In mice, circRNA circSCMH1 presence in EVs has been shown to be a biomarker for ischemic stroke (Yang et al., 2020). Lower levels of circSCMH1 in plasma correlated with a higher chance of stroke in those animals. Further, treatment with circSCMH1 improved recovery after stroke. Defining the role circRNAs play in parasite gene regulation or manipulation of the host immune response is an important next step but will be challenging to accomplish at a technical level. Even in highly tractable model systems, circRNA functionality remains poorly defined for this reason. *In situ* hybridization techniques may allow spatial localization of circRNA and miRNAs of interest in parasite tissues but whilst that might support interactions predicted *in silico*, it may fall short of providing strong functional insight. Several strategies have been used to knockdown expression of circRNAs and provide functional data. Gapmer antisense oligonucleotides can be transfected into cells or tissues of interest to drive RNaseH-mediated cleavage of circRNAs in a sequence-specific manner (Marrosu et al., 2017; Ottesen et al., 2019). Small interfering RNAs (siRNAs) have been used to good effect for downregulating circRNAs in cultured cells (Legnini et al., 2017) and may have some potential for translation to parasitic nematodes as some species are susceptible to RNAi (Song et al., 2010; Verma et al., 2017). Lastly, by targeting back-splice junction sites, a CRISPR/Cas13 approach has been used to successfully screen for circRNA function (Li et al., 2021). This strategy may be possible if DNA transformation of parasitic nematodes becomes more feasible.

Collectively, our data shows that circRNAs are expressed in the parasitic nematode *A. suum* and are also secreted by these parasites in EVs. These findings support the recent description of *H. contortus* circRNAs by Zhou et al. (2021) and better our understanding of how parasitic nematodes may regulate gene expression. Importantly, our recognition that parasitic nematodes secrete circRNAs into the host environment is significant and adds another modality for modulation of host biology to the parasite toolkit. Clarifying the function of these circRNAs will be critical, be they as templates for protein translation or as miRNA sponges. This functional data will provide needed insight into the circRNA-miRNA-mRNA interactome, furthering our understanding of basic parasite biology but may be important for controlling these insidious pathogens. Disrupting circRNA function in the parasite or at the host-parasite interface may help prevent transmission and the establishment of infection.

## Data Availability

Notes and scripts used to produce expression analysis are available at https://github.com/ISUgenomics/Kimber_CircRNA. Raw data from sequencing can be viewed using bio-project number PRJNA750737 with SRA numbers SRR15295818–SRR15295823.

## Supplementary Material

Supplementary Figure S1 1µM IVM, DEC, and LEV do not have an effect on secreted circRNA in *A. suum* extracellular vesicles. Secreted circRNAs were not affected by anthelmintic treatment at 1µM drug treatment after 24 hours. qRT-PCR CT values were normalized to 40ng spike in RNA using 2^−ΔΔCq^. N=3 (minimum). Mean ± SEM, p≤ 0.05 being significant.

Supplementary Figure S2 1µM IVM does not have an effect on circRNA expression in ovary and body wall tissue in *A. suum*. Similar to .1µM IVM treatment, endogenous circRNA expression was not affected by 1µM IVM treatment for either tissue type after 24 hour incubation with drug treatment. qRT-PCR CT values were normalized to 40ng spike in RNA using 2^−ΔΔCq^. N=4 (minimum), Mean ± SEM, p≤ 0.05 being significant.

Supplementary Figure S3 DEC and LEV do not affect circRNA expression in tissues. Single adult female A. suum parasites were incubated for 24 hours in the presence of DEC and LEV at two different concentrations, 0.1µM and 1µM. **(A)** circRNA expression levels DEC treated ovary-enriched and body wall at .1µM and 1µM. DEC did not have any effect on endogenous circRNA expression in ovary and body wall tissues at either concentration. Additionally, LEV treated ovary and body wall tissues did not have significant different in circRNA expression level compared to control at either concentration **(B)**. qRT-PCR CT values were normalized to 40ng spike in RNA using 2^−ΔΔCq^. N=3 (minimum), Mean ± SEM, p≤ 0.05 being significant.

Supplementary Table S1 List of ovary and body wall specific circRNAs.

Supplementary Table S2 Primer table for primers used during qPCR validation. Primer sequence table for circRNA qPCR validation and primers for MEGAScript T7 RNA spike in.
