# Supplementary figures and images for: Expression and Secretion of Circular RNAs in the Parasitic Nematode, Ascaris suum
Source: Front Genet. 2022 May 31;13:884052. doi: 10.3389/fgene.2022.884052 (PMC9194832; doi:10.3389/fgene.2022.884052)

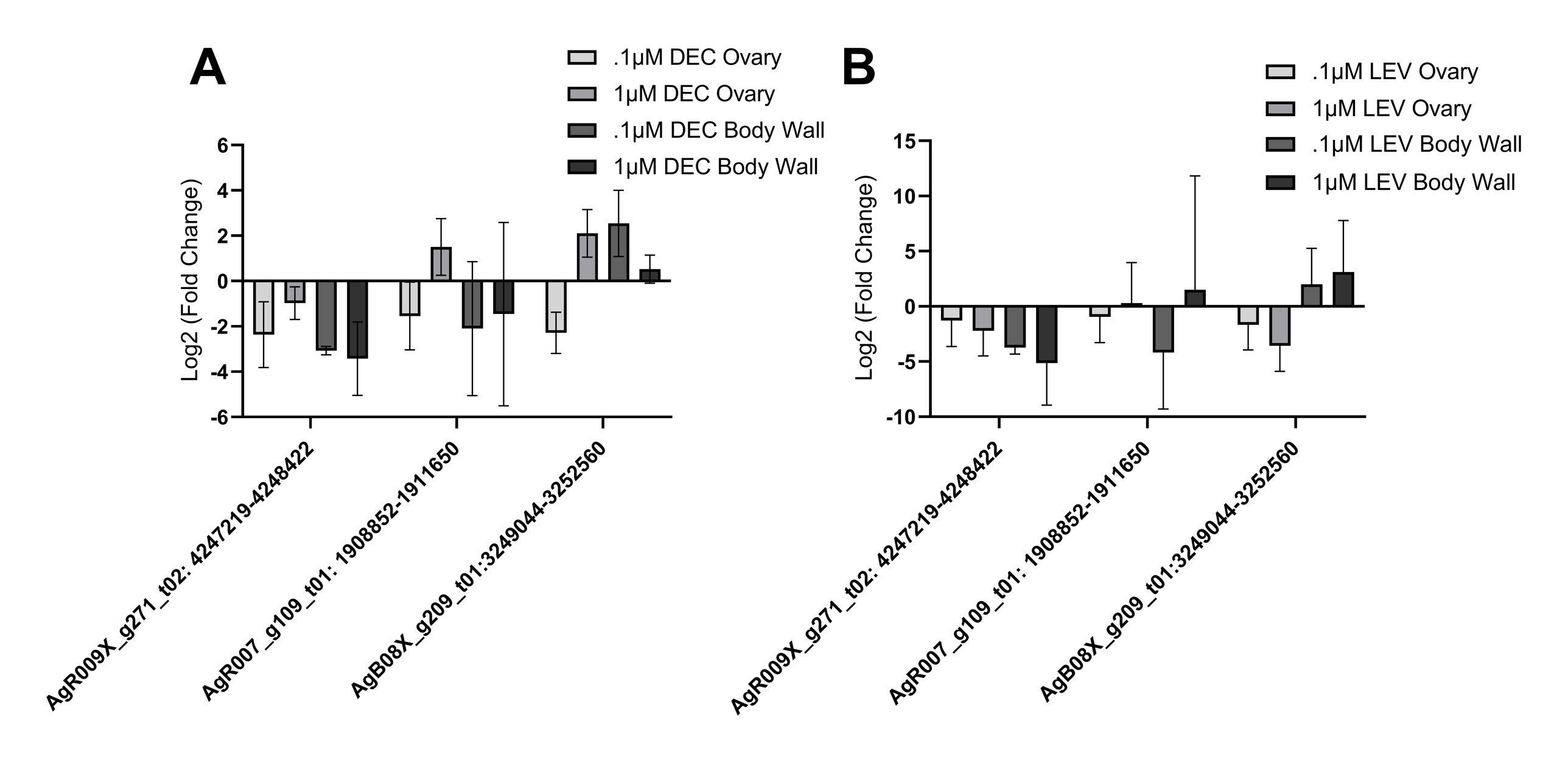

Supplement: Supplementary file 1 [file Image3.tif]

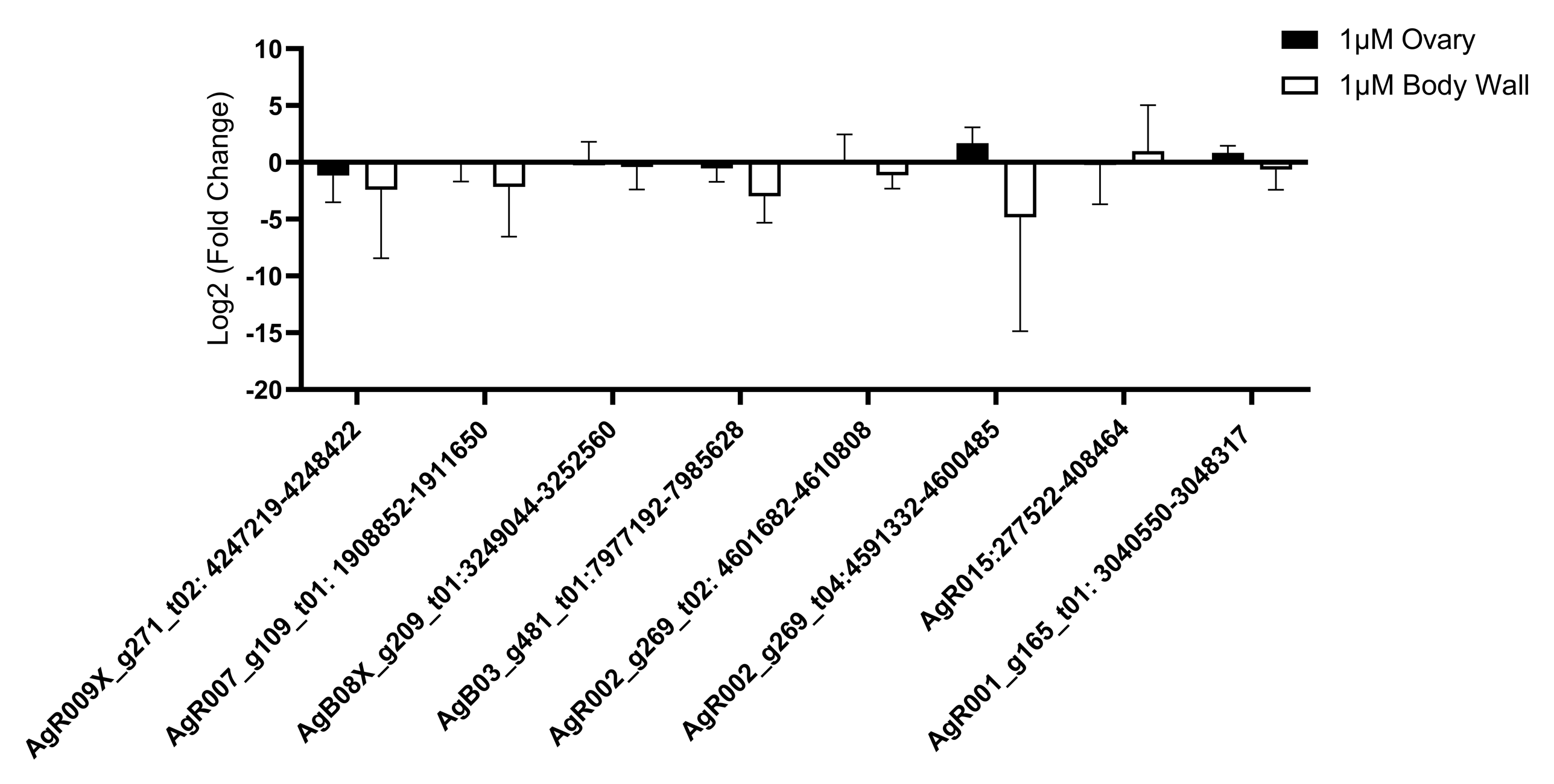

Supplement: Supplementary file 2 [file Image2.tif]

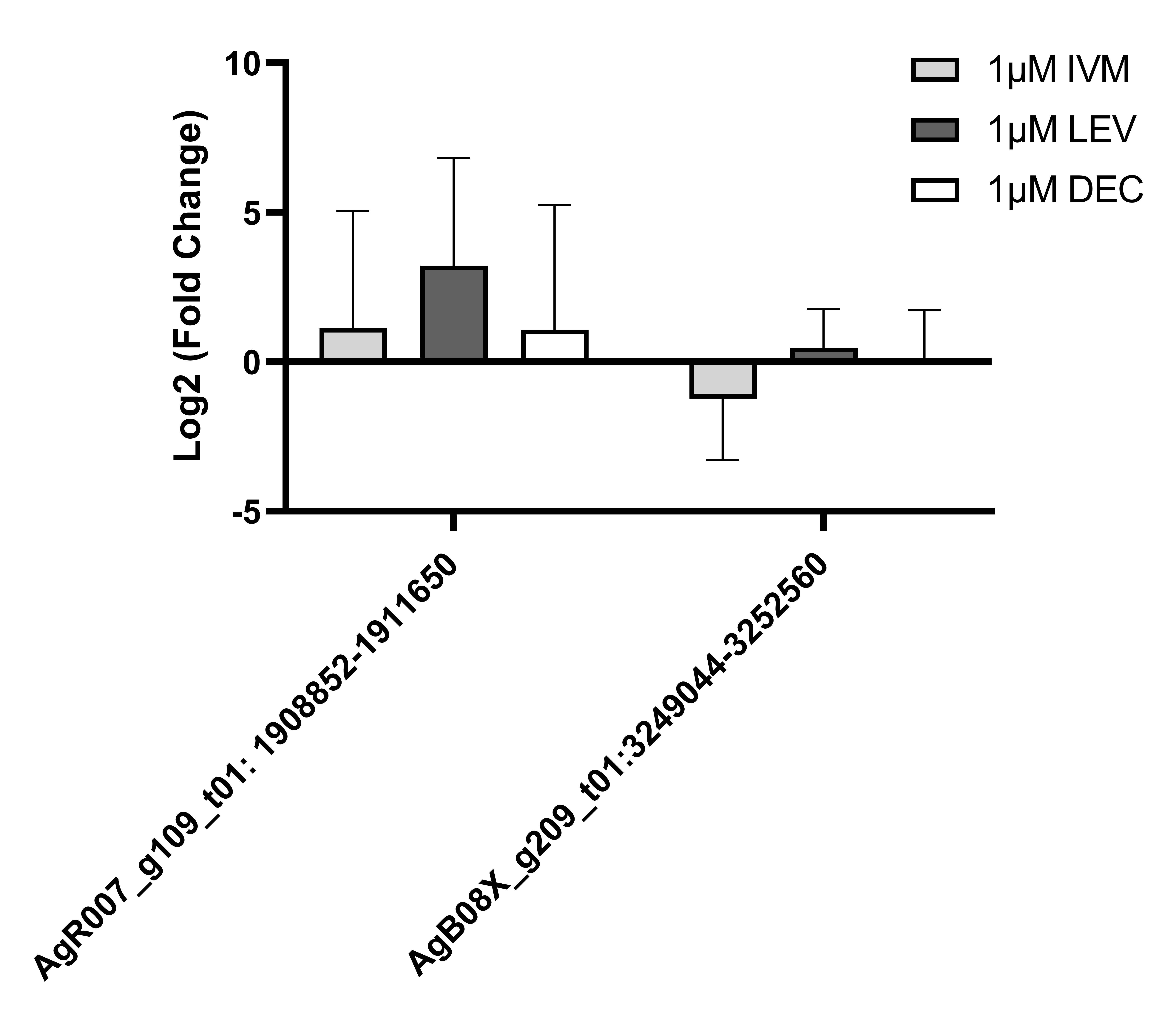

Supplement: Supplementary file 3 [file Image1.tif]
