# Supplementary material for: Expression and Secretion of Circular RNAs in the Parasitic Nematode, Ascaris suum
Source: Front Genet. 2022 May 31;13:884052. doi: 10.3389/fgene.2022.884052 (PMC9194832; doi:10.3389/fgene.2022.884052)
Supplement: Supplementary file 4 [file Table2.docx]

**Supplementary Table 2: Primer table for primers used during qPCR validation**

Primer sequence table for circRNA qPCR validation and primers for MEGAScript T7 RNA spike in.

circRNA circRNA type Primers (5’-3’) Tm (°C)

AgR009X_g271_t02: 4247219-4248422 Exonic F: 5’-AGGGTGACCCTGAACCGCTTAG-3’ 59.2°C

R: 5’-CTTATACTACTTCATGCAC-3’

AgR007_g109_t01: 1908852-1911650 Exonic F: 5’-GTCGTGGGAGAGTACGAGAGC-3’ 58.5°C

R: 5’- GAAACTTGCATTTCCACGC-3’

AgB08X_g209_t01:3249044-3252560 Exonic F: 5’-CGCATTTAATCGTCTCGGTCG-3’ 56°C

R: 5’-CTCACCTTGACACTGGATTCG-3’

AgB03_g481_t01:7977192-7985628 Exonic F: 5’-GTGTAGACGAGGTATTACCAC-3’ 55.5°C

R: 5’- CTCAACGGAGATGTGTGTACCCG-3’

AgR001_g165_t01: 3040550-3048317 Exonic F: 5’-CTAGTTCTCCAAGCAAACAATG-3’ 52.25°C

R: 5’-CAACATTTTTCCCGTCCGC-3’

AgR002_g269_t02: 4601682-4610808 Intronic F: 5’-CTTACCAACTCCAGAAAGC-3’ 55.3°C R: 5’-GTGGCCGATATGGGATCCGG-3’

AgR002_g269_t04:4591332-4600485 Intronic F: 5’- GCGTTCCCGGTACAGACAGAGG-3’ 59.8°C

R: 5’-GAATCGGTTCAGCCGGAGTAG-3’

AgR015:277522-408464 Intergenic F: 5’-GGTGAGACAACCTTCCACC-3’ 55.45°C

R: 5’-CTCATTCGGCTATTTCGTCAG-3’

AgR024_g060_t03: 1256654-1259251 Exonic F: 5’-GGTGTGCACCTACGACAACTAC-3’ 53.5°C

R: 5’-GTAGAAATGAAAAGCACTCG-3’

AgE14_g005_t01: 37677- 38355 Exonic F: 5’-GTACGAAACCGCTTCGAC-3’ 56.2°C

R: 5’-GCCCTCCTTCGACAGCAAC-3’

B-actin Linear RNA F: 5’-CTCGAAACAAGAATACGATG-3’ 57.2°C

(Shao et. al., 2014) R: 5’-CTCGAAACAAGAATACGATG-3’

T7 B-actin Linear RNA

F: 5’ TAATACGACTCACTATAGGGAGATGTCCGTAACAAGACCTGAGAC -3’ 65°C R: 5’-TAATACGACTCACTATAGGGAGACTCATCGTACTCTTGCT-3’
